# Robust Demographic Inference from Genomic and SNP Data

**DOI:** 10.1371/journal.pgen.1003905

**Published:** 2013-10-24

**Authors:** Laurent Excoffier, Isabelle Dupanloup, Emilia Huerta-Sánchez, Vitor C. Sousa, Matthieu Foll

**Affiliations:** 1 CMPG, Institute of Ecology and Evolution, Berne, Switzerland; 2 Swiss Institute of Bioinformatics, Lausanne, Switzerland; 3 Center for Theoretical Evolutionary Genomics, Department of Integrative Biology, University of California, Berkeley, Berkeley, California, United States of America; 4 School of Life Sciences, Ecole Polytechnique Fédérale de Lausanne, Lausanne, Switzerland; University of Washington, United States of America

## Abstract

We introduce a flexible and robust simulation-based framework to infer demographic parameters from the site frequency spectrum (SFS) computed on large genomic datasets. We show that our composite-likelihood approach allows one to study evolutionary models of arbitrary complexity, which cannot be tackled by other current likelihood-based methods. For simple scenarios, our approach compares favorably in terms of accuracy and speed with 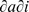, the current reference in the field, while showing better convergence properties for complex models. We first apply our methodology to non-coding genomic SNP data from four human populations. To infer their demographic history, we compare neutral evolutionary models of increasing complexity, including unsampled populations. We further show the versatility of our framework by extending it to the inference of demographic parameters from SNP chips with known ascertainment, such as that recently released by Affymetrix to study human origins. Whereas previous ways of handling ascertained SNPs were either restricted to a single population or only allowed the inference of divergence time between a pair of populations, our framework can correctly infer parameters of more complex models including the divergence of several populations, bottlenecks and migration. We apply this approach to the reconstruction of African demography using two distinct ascertained human SNP panels studied under two evolutionary models. The two SNP panels lead to globally very similar estimates and confidence intervals, and suggest an ancient divergence (>110 Ky) between Yoruba and San populations. Our methodology appears well suited to the study of complex scenarios from large genomic data sets.

## Author Summary

We present a new likelihood-based method to infer the past demography of a set of populations from large genomic datasets. Our method can be applied to arbitrarily complex models as the likelihood is estimated by coalescent simulations. Under simple scenarios, our method behaves similarly to a widely used diffusion-based method while showing better convergence properties. In addition, our approach can be applied to very complex models including as many as a dozen populations, and still retrieve parameters very accurately in a reasonable time. We apply our approach to estimate the past demography of four human populations for which non-coding whole genome diversity is available, estimating the degree of European admixture of a southwest African American population and that of a Kenyan population with an unsampled East African population. We also show the versatility of our framework by inferring the demographic history of African populations from SNP chip data with known ascertainment bias, and find a very old divergence time (>110 Ky) between Yorubas from Western Africa and Sans from Southern Africa.

## Introduction

Reconstructing the past history of a given species is important not only for its own sake, but for disentangling demographic from selective effects [1], [2]. Demography is indeed often estimated on a set of markers and the best neutral model is used as a null for evidencing markers under selection [3], [4] or for finding global patterns of selection across the genome [e.g. 5]. Various methods have been proposed to estimate demography from genetic data, including full-likelihood methods [6]–[9], summary-statistics likelihood based methods [10], [11], or different flavours of Approximate Bayesian Computation [12]–[16]. With some exceptions, these methods are relatively slow and do not scale up very well with new genomic data, as computation time increases with the number of loci. In contrast, recently developed composite-likelihood methods based on the site frequency spectrum [SFS, 17] have computing times that do not depend on the amount of available genomic data [18]–[21], and several approaches have been proposed to estimate demographic parameters from the SFS [e.g. 11], [17], [20], [21]–[24]. Among these latter methods, the most widely used is 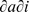 [21], which estimates the expected joint site frequency spectrum for an arbitrary set of parameters by a diffusion approach. Whereas the estimation of the expected SFS is relatively fast, the optimization of the parameters is still time-consuming, which prevents 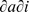 to tackle models with more than three populations at the same time. While some methods can extract demographic information from single whole-genomes per population [25], [26], SFS-based methods, when applied to multiple individuals, do not require whole genome data because correct estimates of the SFS can be obtained from a few Mb [21]. However, with few exceptions [11], the accuracy of SFS-based methods has not been properly assessed, and their ability to infer demographic parameters has been questioned [27].

One advantage of SFS-based inference methods is that they can handle large next generation sequencing (NGS) data sets [28]–[30]. However, the computation of the SFS from NGS data is not always trivial. An empirical Bayes approach has been proposed to estimate the joint 2D SFS from low coverage data [31] and an unbiased maximum likelihood approach has been developed to recover the SFS for a single population [32]. SFS obtained from low-coverage genomic data often show a deficit of rare alleles because a given allele needs to be observed in several individuals to exclude read errors [28], [33]. These missing low frequency variants can lead to imprecisions and biases in population genetic inferences [34]. Several approaches have been proposed to correct for this bias [32], [35], either during the process of genotype calling itself [e.g. 31], [36], [37] or later by applying quality filters on called genotypes [e.g. 38]. Gravel et al. [28] have also proposed to predict the SFS from low-coverage data by using an overlapping subset of high quality data to derive a generalized correction of the SFS. It appears likely that SFS estimation will improve with higher coverage NGS data, and that such data will become increasingly available and used in the near future.

As an alternative to deep sequencing, one could use information from a few tens of thousands SNP scattered over the whole genome to make demographic inference, but most SNP chips have complex and often unknown ascertainment schemes that bias the SFS if not properly taken into account [39]–[41]. However, a new SNP chip has recently been introduced [42], [43], which implements a known and simple ascertainment scheme where SNPs are selected at random from sites that are heterozygous in a single individual of a given population. Whereas this ascertainment scheme has no major effect on statistics designed to infer admixture [42], it biases the site frequency spectrum [44], [45] and thus potentially alters the estimation of other parameters. Using simple combinatorics, the SFS can be unbiased [44] in a single population, and this strategy could be extended to unbias joint SFS under complex models involving more populations. A diffusion approach has been recently proposed to estimate divergence times between two populations based on the fraction of SNPs having occurred recently in the ascertained population [45], but this approach is currently restricted to the sole estimation of divergence time and cannot be applied if gene flow occurred between populations.

In this paper, we introduce a flexible and robust way to estimate demographic parameters from the SFS inferred from sequence or SNP chip data that we implemented in the *fastsimcoal2* software. Our method is based on Nielsen's approach [17], which estimates the expected SFS from simulations under any demographic model. We compare the performance of this approach to 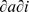 [21] under a variety of evolutionary models with simulated data, and we show that it can successfully handle models including more than three populations. We also show how this approach can be extended to deal with ascertained SNP panels by explicitly modelling the ascertainment bias and computing likelihoods based on expected ascertained SFSs. We first apply our method to a large human genomic data set from which we estimate the demography of four populations, and then to two separate Affymetrix ascertained SNP panels [43] from which we estimate the demography of two African populations.

## Results

### Comparison between *fastsimcoal2* and 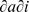


**Figure 1:**
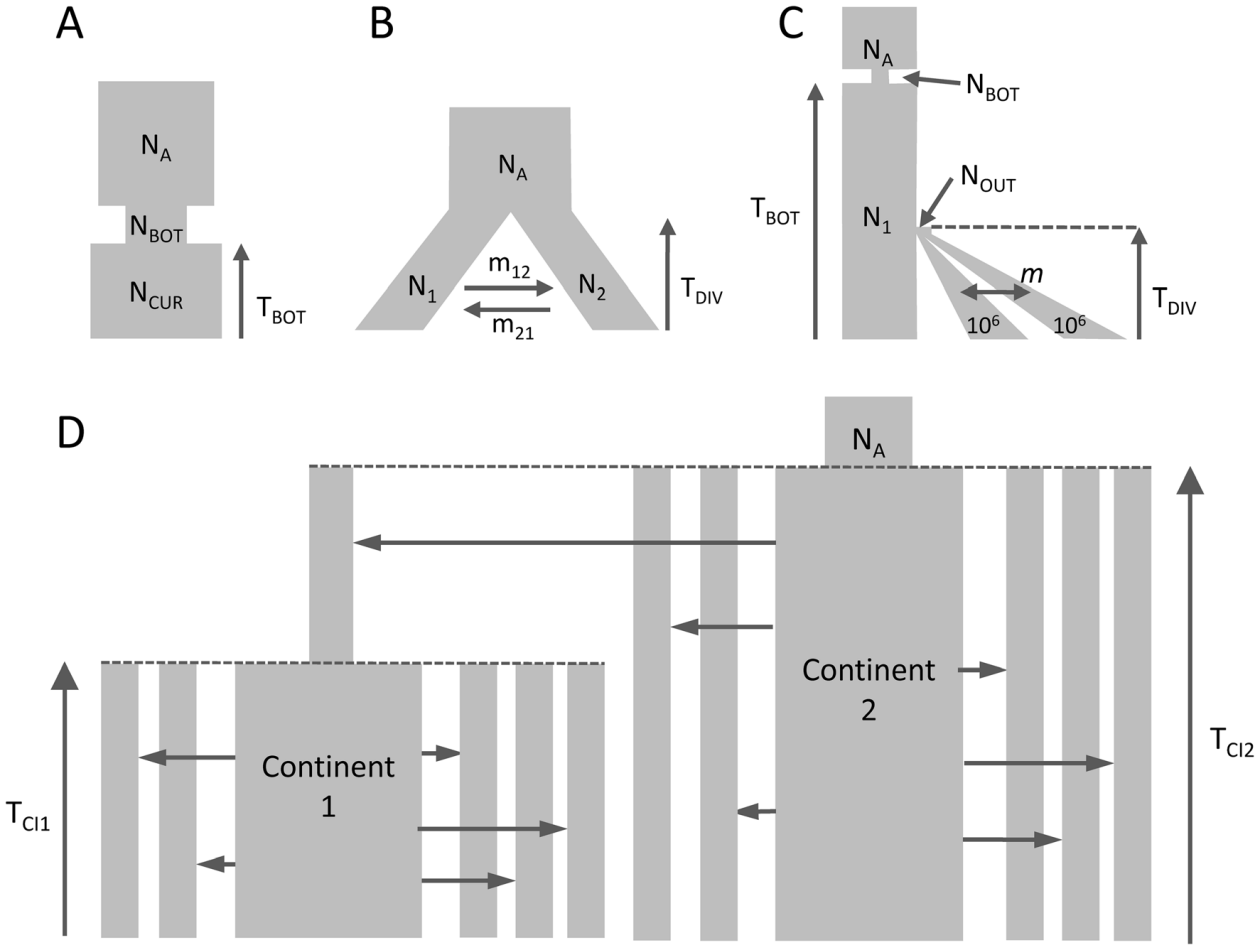
Tested demographic models. A) One population with bottleneck B) Isolation of two populations with asymmetric migration C) Three population divergence with migration and bottleneck. This model corresponds roughly to a model of human differentiation, where N_1_ would be the size of an African population, and T_DIV_ would correspond to the exit of a population diverging into Asian and European populations growing exponentially and still exchanging migrants at rate m. We assume that the current size of the expanding population is known and equal to 1 million diploids. D) Divergence of two continent-islands. We assume that two Continent-Island systems were created *T*_CI1_ and *T*_CI2_ generations ago, with the youngest continent stemming from one of the island of Continent 2. The parameters of interest are the per generation number of migrant genes (*M* = 2*Nm*) from each continent to each island, the age of the continents and the ancestral population size *N*_A_. The island population sizes were set to 500 diploids and *M* changed due to immigration rates *m* that could differ for each island.

We performed parameter estimations for 10 data sets generated under each of the 3 evolutionary scenarios shown in Figures 1A–1C. We took two approaches for estimating demography: our new approach based on a composite multinomial likelihood where the expected SFS is obtained using coalescent simulations and 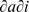 [21], which computes a composite Poisson likelihood where the expected SFS is obtained by a diffusion approximation. The two approaches have a very similar accuracy under a simple bottleneck scenario (Figure S4) and under a scenario of population isolation with migration [46] (IM model, Figure S5). For both approaches we report the estimates leading to the maximum likelihood obtained among 50 independent runs. Under these conditions, 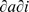 leads to extremely accurate estimations for most data sets. However, in a few cases (1/10 for the bottleneck scenario, and 2/10 for the IM model), the best likelihood obtained from 50 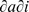 runs led to very divergent estimates, which were not reported in Figures S4, S5. For those cases, the log likelihood appeared orders of magnitude smaller than those inferred for other data sets and could be easily spotted. Although it is possible to recognize that additional 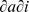 runs are necessary to get meaningful estimates, we did not follow this procedure here, as we wanted to allocate similar resources to the two programs and get results using an automated procedure not requiring further user tweaks. Contrastingly, *fastsimcoal2* estimations seem to converge to correct values for all data sets in Figure S4 and S5, even though the variances of the estimators are slightly larger than 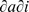
's for those cases where both approaches agree on the correct demographic model.

**Figure 2:**
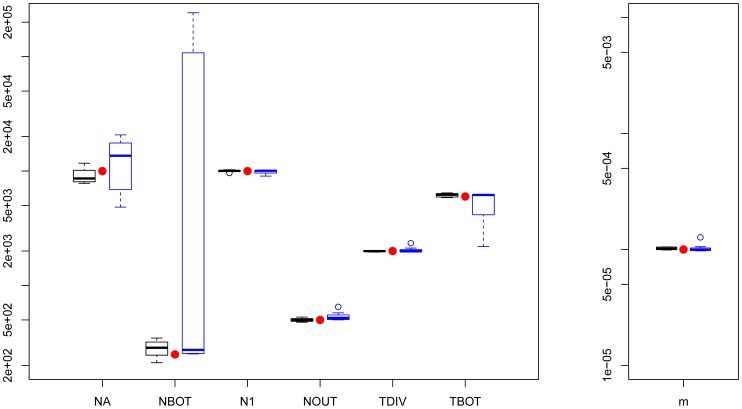
Three population divergence and growth model. *fastsimcoal2* results are in black and 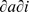
's results (8/10) are in blue. True parameters values are shown as red dots. *fastsimcoal2* required 4–5 h for a single estimation based on 40 ECM cycles over parameters, whereas a run of 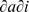 requires on average 34 hours on a similar CPU.

Parameter estimations under the more complex scenario of Figure 1C, mimicking a simple model of human evolution, are reported in Figure 2. In this case, results obtained by *fastsimcoal2* are again very accurate and close to the true values for all 10 data sets. With 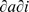, we report results for only 8 data sets due to potential lack of convergence, as explained above. However, even for these 8 data sets, the best estimates can be quite far from the true parameters, especially for parameters related to the ancestral bottleneck. It suggests that for complex scenarios involving three populations and more than 5 parameters, 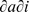 needs to be run from many more than 50 initial conditions and that some iterative refinements of search ranges might be necessary to obtain correct solutions (R. Gutenkunst, personal communication). Note that a lack of robustness of 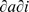 under certain conditions (e.g. high migration rates between populations) had already been reported before [11], [24].

### Estimation of parameters under a scenario with more than 3 populations

**Figure 3:**
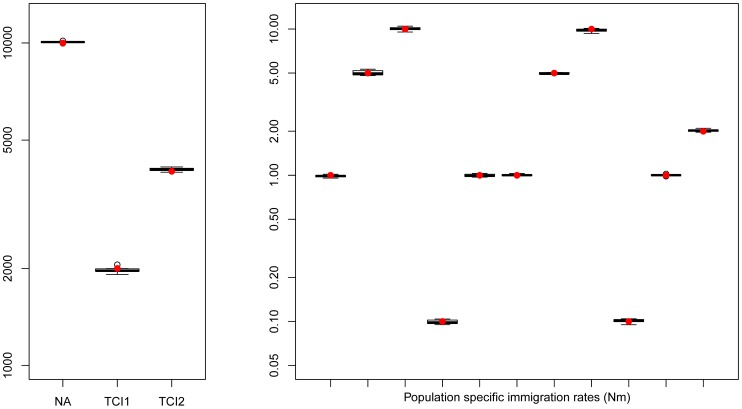
Hierarchical islands model. Boxplots showing the distribution parameters estimated from 10 data sets simulated under the same scenario. True parameter values are shown as red dots. *fastsimcoal2* required 35–40 hours for a single estimation based on 30 ECM cycles over parameters, using 50 thousand simulations to estimate the expected SFS under a given set of parameters.

We have estimated parameters for the more complex hierarchical continent-island model shown in Figure 1D, involving samples from 10 different populations (islands), a model that 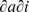 cannot handle. Continent-island models are equivalent to infinite islands models, and have been used to model recent spatial expansions [see e.g. 47]. This model could therefore represent two successive spatial expansions, the first one stemming from an ancestral refuge area, and the second one starting more recently from a single deme belonging to the first expansion wave. The parameters of interest are here the immigrations rates in each sampled deme, the timing of the spatial expansions and the ancestral population size. As shown in Figure 3, all these parameters are extremely well estimated by *fastsimcoal2* when we maximize the multiple pairwise composite-likelihood shown in eq. (7). We note that we can also recover very well the immigration rate to the unsampled deme (rightmost column in Fig. 3) from which the second expansion started. The accuracy of the immigration rate estimations is quite remarkable, given that they span over two orders of magnitude and that we specified the same search intervals covering four orders of magnitude for each parameter.

### Estimation of human demography from non-coding genomic data

**Figure 4:**
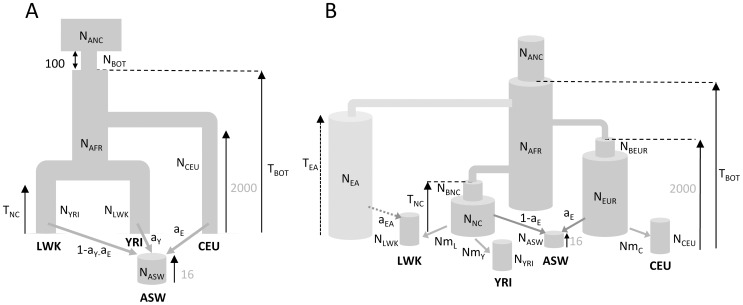
Demographic models of four human populations. A: Simple model of African American (ASW) admixture supposed to have occurred 16 generations ago, with contributions from 3 potential sources (Europeans : CEU; Yoruba: YRI; Luhya: LWK. The European population is assumed to have diverged 2000 generations ago (50 Kya, [28]) from Africa. B1: More realistic demographic scenario (dark grey) of African American admixture and population differentiation, based on continent-island models used to depict spatially arranged populations after range expansions [see e.g. 47]. B2: same as B1 but with an additional possible admixture of Luhya from an unsampled (possibly East African) population. The extra parameters and population of model B2 are shown with a lighter shade of gray and with dashed arrows, respectively. The models and their parameters are further described in the Material and Methods section.

We first applied our methodology to the problem of estimating the past demography of two African, one European and one African-American populations. The multidimensional SFS for these 4 populations was estimated from more than 220,000 non-coding SNPs, each located more than 5 Kb away from its closest neighbour, such as to minimize linkage disequilibrium between SNPs. We examined three evolutionary scenarios shown in Figure 4 to explain observed patterns of diversity. In the first and simplest scenario (Figure 4A), the South Western African American population (ASW) was assumed to have been formed 16 generations ago (around 1600 AD) with initial input from one European (CEU) and two Niger-Congo speaking African populations (Yoruba from Nigeria: YRI; Luhya from Kenya: LWK) having diverged earlier. In order to calibrate the other parameters, we assumed that the European population diverged from the ancestral African population 50 Ky ago [28], [48]. Under this scenario, we find that the ASW population would have initially received 16% (CI95% = [15–17%]) of its gene pool from the CEU population, 83.8% from the YRI population and almost nothing (0.2%) from the LWK population (see Table 1, Model A). This European contribution is in line with previous estimates obtained from SNP-chip allele frequencies (17% for Southwest African Americans [49]). Under model A, the two Niger-Congo populations would have diverged very recently (70 generations ago, CI95% = [56–197]), and the CEU and YRI populations have the smallest effective population sizes (around 4000 individuals), whereas the ASW population has the largest (N_ASW_ = 170,000 individuals). The inferred human ancestral population size is relatively small (about 8000 individuals) and there is no real signal of an ancestral bottleneck since the estimated bottleneck size (N_BOT_ = 7083) is only 12% smaller than the ancestral size, in line with recent results showing no evidence for a strong Pleistocene bottleneck in humans [50].

**Table 1:** Inferred parameters of human demography under model B1 and B2 defined in Figure 4B.

	Model B1			Model B2		
	Point estimation	95% CI^a^		Point estimation	95% CI^a^	
Parameters		Lower bound	Upper bound		Lower bound	Upper bound
N_ANC_	13405	12075	15923	12386	10986	14875
N_AFR_	27519	23246	38250	25536	22054	35939
N_ASW_	38287	10470	41812	9219	9906	44026
N_CEU_	27070	3673	44075	38623	8842	43883
N_LWK_	26793	15395	44540	10711	13288	41103
N_YRI_	6635	5546	12003	22835	14809	44010
N_EUR_	16689	12818	40709	14530	11792	25615
I_BEUR_ b	0.432	0.395	0.472	0.418	0.375	0.450
N_NC_	164535	41032	401691	56697	33872	414434
I_BNC_ b	0.026	0.019	0.071	0.027	0.011	0.040
2Nm_C_	2.08	0.03	13.56	0.05	0.04	26.57
2Nm_Y_	8.66	0.04	19.37	0.52	0.04	22.83
2Nm_L_	10.93	0.03	29.40	5.18	0.03	35.68
T_NC_	793	567	1814	797	509	1981
T_BOT_	10059	8526	12932	9971	8900	12834
a_E_	0.16	0.15	0.18	0.17	0.16	0.18
N_EA_				228516	95844	451516
T_EA_				2230	1479	3386
a_EA_				0.17	0.08	0.19

a Parametric bootstrap estimates obtained by parameter estimation from data sets simulated according to CML estimates shown in the point estimation column.

b Bottleneck intensity is equal to bottleneck duration (100 generations) divided by the bottleneck population size (N_BEUR_ or N_BNC_).

Conditions for *fastsimcoal2* point estimations were: 50–250,000 simulations per likelihood estimation (-n50000, -N250000), 30 ECM cycles (-L30), 50 runs per data set. Conditions for *fastsimcoal2* CI estimations were: 100,000 simulations per likelihood estimation (-N100000), 30 ECM cycles (-L30), 10 runs per data set.

Whereas model A captures some obvious features of the past demography of these populations (see Table S1), it seems relatively unrealistic for some other features (i.e. a direct contribution of the CEU and YRI populations to ASW). We therefore investigated a more realistic but more complex and parameter-rich model involving several other unsampled populations, as shown in Figure 4B (see Material and Methods for a complete description of this model). The multiple continent-island model B1 assumes that the ASW population was founded by migrants originating from a Niger-Congo and from a European metapopulations, from which the two Niger-Congo and the CEU populations currently receive migrants. It also assumes that the Niger-Congo and the European metapopulations passed through a bottleneck when they diverged from an ancestral African population. An even more complex scenario B2 includes a potential admixture of the Luhya population (a Niger-Congo speaking population from Kenya) with an unsampled (potentially East-African) population, which also diverged earlier ago from the ancestral African population.

The model parameters estimates and their confidence intervals obtained by a parametric bootstrap approach are listed in Table 1. The two models show overall very congruent values and overlapping 95% confidence intervals for their common parameters. The agreement is especially good for the human ancestral size (N_ANC_ = 12–13,000 individuals), the ancestral African population size (N_AFR_ = 25–27,000), the continental European size (N_EUR_ = 14,500–16,500 individuals), the European strong bottleneck intensity (I_BEUR_ = 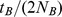 = 0.42–0.43, where 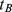 is the bottleneck duration, and 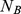 is the bottleneck size), the Niger-Congo milder bottleneck intensity (I_NC_ = 0.027–0.028), the divergence time of the Niger-Congo metapopulation (T_NC_ = 793–797 generations), the time to the shift to the ancestral human population size (T_BOT_∼10,000 generations), and the European contribution to the ASW population (a_E_ = 0.16–0.17). The other parameters show different point estimates but all have overlapping confidence intervals.

We have plotted the marginal SFS for each of the four populations in Figure S6, to visualize the fit of the expected and observed SFS for each model. Whereas the expected population specific marginal SFSs show some discrepancies with the observation for the four populations under model A, the fit is much better for model B1, except for LWK, which still shows an underestimation of singletons and doubletons. Model B2, which allows for LWK admixture, leads to a much better fit for the LWK population, as shown by the cumulative distribution of differences between the expected and observed marginal SFS (see 3^rd^ row in Figure S6). Under this model B2, we estimate the LWK population to have 17% admixture from an unspecified but probably East African (see e.g. Figure 1 in ref. [51]) population. This East African population would have diverged from the ancestral African population more than 2200 generations ago (95% CI 1274–3586), thus potentially before the out-of-Africa dispersal. Even though the different models can be conveniently compared on the basis of their marginal SFSs, these 1D SFSs only capture a small fraction of the total (multidimensional) SFS. Therefore the models are better compared on the basis of their likelihood. This is formalized here by a model comparison procedure based on AIC [52], revealing that the relative likelihood 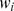 of models A and B1 are almost 0 as compared to that of model B2 (see Table S2).

### Estimation of African demography from ascertained SNP panels

**Figure 5:**
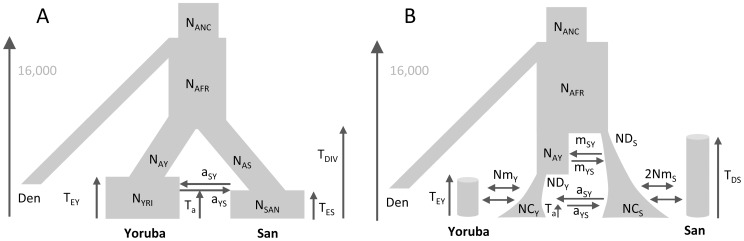
Alternative demographic models for two African populations. A) Simple model of population divergence. The San and Yoruba populations are assumed to have split from an ancestral African population and to have gone through a recent populations size increase. They also had a single pulse of asymmetrical gene flow (admixture) T_a_ generations ago. B) More complex scenario, where the San and Yoruba demes belong to two distinct continent-island structures, which have also admixed asymmetrically T_a_ generations ago. The ancestral Yoruba and San populations would have gone through exponential growth at different times, and have exchanged genes just after their divergence until T_EY_ generations ago. In both models, we assumed that the Denisova population diverged from the ancestral human population 16,000 generations ago, as estimated in [58] based on an ancestral population size of 10,000 diploids (see Table S11.2 in Suppl. Mat. of ref. [58]). This date would correspond to ≈400,000 years assuming a generation time of 25 y. The models and their parameters are further described in the Material and Methods section.

We estimated the parameters of African past demographies shown in Figure 5 based on Yoruba and San samples for which we have independent SNP panels (see Methods section). In model A (shown in Figure 5A), we assumed that the Yoruba and San samples were taken from large populations that expanded after their divergence, and we allowed for a single pulse of gene flow between them at a given time *T_a_* in the past. The model B (shown in Figure 5B) includes the divergence of two-continent island metapopulations, and assume that the sampled populations are each an island attached to these continents and that the two continents exchanged migrants some time ago in a single pulse of gene flow, like in model A, but also earlier in time (see Figure 5B and material and methods for a complete description of the model).

The point estimates of the two models and their associated 95% confidence intervals (CI) inferred from 100 parametric bootstraps are reported in Table 2 for both SNP panels. Overall, the two SNP panels show congruent point estimators and CI widths under the two models. There is only one parameter (N_AY_) for which the CI do not overlap under model A, which suggests that the two panels provide broadly compatible scenarios of African demography. Estimations from data simulated under the same model for parameter values similar to those inferred in Figure 5A show (see Figure S8) that i) both panels should perform very similarly for estimating parameters, ii) all parameters of the model should be well estimated, except those related to a very recent expansion of one of the ascertained population, iii) ancestral population sizes and divergence times are particularly well estimated, and iv) the addition of a single Denisovan sequence allows one to recover the absolute values of the parameters.

**Table 2:** Inferred parameters of African demographic history for model A and B.

Model A					Model B				
	Panel 4 (San)		Panel 5 (Yoruba)			Panel 4 (San)		Panel 5 (Yoruba)	
Parameters	Point estimation	95% CI^a^ ^b^	Point estimation	95% CI^a^ ^b^	Parameters	Point estimation	95% CI^a^ ^b^	Point estimation	95% CI^a^ ^b^
N_ANC_	9680	9300–10700	9017	8500–9900	N_ANC_	9612	8977–10424	9013	8384–10146
N_AFR_	27835	26400–33500	17746	16800–20700	N_AFR_	23849	21634–44081	21762	15867–46813
N_SAN_	741378	166700–811800	532609	139900–873500	NC_S_	180,771	16598–411442	224,695	38694–446151
N_YRI_	517893	106600–890100	463052	124200–941000	NC_Y_	96,071	2464–461785	251,150	67722–428360
N_AS_	8473	2400–10300	11140	8900–13900	ND_S_	3,704	412–6996	5187	2,000–5,700
N_AY_	5528	2000–7800	13087	11200–14800	N_AY_	10251	2456–461785	5480	1730–15823
					ND_Y_	644	85–4553	3654	517–4680
					2Nm_S_	5.9	4.6–14	3.7	3.4–18
					2Nm_Y_	37.4	5–77	36.8	25–88
T_a_	523	271–766	650	310–868	T_a_	59	10–100	77	16–95
a_YS_	0.16	0.04–0.22	0.19	0.12–0.22	a_YS_	0.19	0.04–0.28	0.08	0.03–0.19
a_SY_	0.11	0.06–0.15	0.10	0.06–0.13	a_SY_	0.08	0.04–0.18	0.16	0.06–0.25
					m_SY_	4.45E-05	2.3E-06–9.9E-04	2.56E-04	3.1E-06–1.0E-03
					m_YS_	1.11E-04	1.2E-05–6.3E-04	1.53E-04	6.2E-06–2.4E-04
T_EY_	32	20–260	113	38–249	T_EY_	170	101–691	298	162–567
T_ES_	63	54–480	232	73–401					
T_DIV_	2599	999–3834	4689	3716–5013	T_DS_	5,530	2482–9710	10,330	5358–12561

Parameters are defined in Figure 5.

a Population size estimates have been rounded to the nearest hundred.

b Parametric bootstrap estimates obtained by parameter estimation from 100 data sets simulated according to CML estimates in point estimation columns.

Estimates were obtained from 100,000 simulations per likelihood and 20 ECM cycle.

Concentrating on the parameters common to both models, we see in Table 2 that the ancestral size N_ANC_ shows very similar estimates across models and panels, with an estimated value around 9,000–9,500 individuals (in line with estimates obtained with non-ascertained data set). The African population size is also consistently estimated to be around 18,000–28,000 individuals across models, and the ancestral Yoruban size appears smaller and between 5,500 and 13,000 individuals. These estimates fit well with previous Bayesian estimations of African demography from nuclear markers under slightly different models. Based on microsatellites, Wegmann et al. [13] estimated the ancestral size of Niger-Congo (NC) populations (to which Yoruba belong) to be 12,500 individuals and that of the ancestral African population to be 15,000 individuals. More recently, the analysis of 40 non-coding regions of 2 Kb [53] led to estimates of NC and African ancestral size to be 17,500 and 11,000 individuals, respectively, as well as a San effective size of the order of 20,000 individuals. The differences between these estimations and ours might be due to the fact that these previous analyses were based on slightly different models that assumed constant sizes for all current populations and the same population size before the split with Denisovans.

In addition, we find evidence for some asymmetrical gene flow between San and Yoruba, around 500–600 generations ago (12.5–15 Kya) under model A, and much more recently (60–80 generations ago) under model B. Interestingly, this is the only parameter common to the two models that shows such drastic difference. Despite this disparity, which could be due to the fact that we allow for earlier migration between the two metapopulations in model B, we obtain very similar estimates for the admixture rates between populations both between panels and across models. Overall, we find a slightly larger extent of gene flow from Yoruba to San than the reverse, but the confidence intervals of the two parameters seem quite overlapping under both models. Under model A, the point estimates for the divergence time T_DIV_ are much more different than what was obtained under our simulations (Figure S8), with a much younger divergence suggested by the San panel (2,600 generations or 65 Kya) than for the Yoruba panel (4,700 generations or 117.5 Kya). Taking the middle of the overlap between the two CI would lead to a divergence time of 4,500 generations or 112.5 Kya (Table 2), in keeping with a recent estimate of the divergence of Khoisan populations obtained by an ABC approach [110 Ky, 53], and compatible with the divergence time estimated between San and other West African population (65–120 Ky in [54], or ∼100 Ky in [55]). Under model B, the two estimates obtained for panel 4 and 5, show a similar discrepancy, but the estimated values are much higher (5,530 and 10,330 generations for panels 4 and 5, respectively), which can also be due to the fact that we authorize some gene flow between the two metapopulations after their divergence. If we again take the middle of the overlap between the two CI, we obtain a value of 7,500 generations (180 Kya), substantially larger than the value obtained under model A (4,500 generations).

An examination of the parameters restricted to model B suggests that the Yoruban continent expanded recently 170–300 generations ago (4250–7500 ya), from a relatively small population of 600–3600 individuals, and that the Yoruban island receives more migrants (around 18 per generation) than the San island (2–3 individuals per generation). The age of the expansion is slightly older than the divergence time between two Western Niger-Congo populations estimated previously (140 generations, [13]), and intermediate between the age of the Niger-Congo languages (∼10 Kya, [56]), and that of the Bantu expansion (∼5 Kya, [57]). The larger immigration rate seen in Yorubans is compatible with the fact that farmer populations generally maintain higher levels of gene flow with their neighbours than hunter-gatherers due to their larger effective size [47]. Note however that all parameter estimates mentioned above assume that the Denisova divergence time is correctly estimated at 16,000 generations or 400 Kya [58], even though there is still a large uncertainty attached to this divergence time, which could range from 230 to 650 Kya [58] or even between 170 and 700 Kya in a more recent study [59]. Reported estimates and CI in Table 2 do not take this uncertainty into account, and should thus be rescaled if a different divergence time between Denisovans and Humans was proposed.

Like in the case of non-ascertained data, we find that the more complex model is much better supported by the data. Even though this better fit is barely visible when considering the marginal 1D expected SFS (see Figure S10), this is more exactly quantified by an AIC analysis (Table S3) revealing that the relative likelihood of model A is close to zero for both panels when compared to model B.

## Discussion

### Estimation of demographic parameters from genomic data

We have introduced a new and flexible simulation-based approach to estimating demographic parameters. For the tested scenarios, our composite-likelihood approach is as precise as 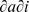 [21], which is the current standard in the field. Our approach seems more robust than 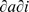 since it is more likely to converge towards the correct solution when starting from the same number (50) of initial conditions (see Figures 2, 3, S4, S5). In terms of computational speed, point estimates are very quickly obtained by 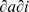 for simple models (on average 15 seconds and 6 minutes for models in Fig. 1A and 1B, respectively, compared to 15 minutes and 2h30 for *fastsimcoal2*, respectively). However, *fastsimcoal2* is much faster for more complex models with three populations and migration (4–5 h per run for *fastsimcoal2* for model on Fig. 1C, compared to 34 h on average for 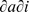). By maximizing the fit of two-dimensional SFS, *fastsimcoal2* can also explore very complex models involving more than 10 populations with migration, which cannot be tackled by any other current method. Since fastsimcoal2 and 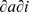 use a very similar likelihood function (see Figure S3), it seems that the improved convergence of our approach lies in the use of the ECM optimization scheme, which compensates for the use of non-optimal approximate likelihoods. Note that our robust ECM maximization technique and the maximization of the product of pairwise composite likelihoods could also be used by methods deriving the SFS analytically or by a diffusion approximation (like 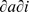), thus potentially enabling the analysis of models as complex as those studied here. Also note that recent progress in the computation of joint SFS using coalescent or diffusion approaches [18], [23] have led to the development of promising demographic inference methods applied to the study of relatively complex evolutionary models [see e.g. 24].

Even though different demographic trajectories can lead to exactly the same SFS in a single population [27], we do not find any evidence of parameter non-identifiability in our investigated cases. This is probably because we restricted our search to a limited set of possible histories, defined by few-parameter models. Our results confirm that if the true history lies within the models considered, the parameters of relatively complex scenarios can be well recovered from the (joint) SFS. However, we must keep in mind that histories outside our model family might have identical likelihoods.

One disadvantage of our method (and of any other simulation-based method) is that we are approximating the likelihood, implying that two runs from identical initial parameter values can results in different estimations (see Figure S2). Using more simulations for the estimation of the likelihood would lessen but not totally suppress this problem, but our results show that our maximization procedure leads to almost completely unbiased estimates and converges to correct values. Another disadvantage of our approach is its dependence on composite likelihoods. More powerful full likelihood approaches explicitly take into account linkage disequilibrium (LD) between sites [60], and therefore might reveal useful to infer recent migration events (see e.g. [61]). That being said, our applied data sets consist of SNPs randomly distributed across the whole genome, and so patterns of LD between sites are minimal. Whereas confidence intervals of demographic parameters based on composite likelihood ratios should in principle be too narrow (see e.g. [21], [60], [62], [63]), a study based on short stretches of DNA sequences has empirically shown that they were extremely similar to those obtained by explicitly modeling patterns of recombination [54]. This appears unlikely to be true in general, and certainly not if products of pairwise composite likelihoods were used (as with eq. (7), which was actually not used for our test cases). Similarly, the use of composite likelihoods in model tests based on AIC can overestimate the support for the most likely model [64]. However, the composite likelihoods in our test cases are quasi likelihoods due to the global independence between SNPs, and the differences in relative likelihood of alternative models are so huge (see Tables S2 and S3) that some residual patterns of LD are unlikely to change our conclusions.

As an alternative to our composite likelihood maximization approach, Garrigan [22] has proposed to integrate an approximate likelihood computed in a way similar to ours into an MCMC algorithm, allowing him to get posterior distributions and credible intervals. Whereas MCMC algorithms generally assume that the likelihood is computed accurately, it has been shown that MCMC procedure should lead to correct posterior distributions even if the likelihood is approximated, provided that there is no systematic error in its computation [65], [66]. This Bayesian approach could be worth exploring as a possible extension of our likelihood maximization procedure. However, our current implementation has the advantage of quickly getting point estimates, around which CIs can be obtained later by repeating the estimation on bootstrapped samples. For instance, a point estimate for the IM model shown in Figure 1B is obtained in about 2h30 on a single core machine, whereas 40–80 h are necessary to get posterior distributions for the parameters of a similar IM model from a single MCMC run using a specialized coalescent program on a multi-core machine [see 22].

### Handling ascertained SNP data sets

The additional versatility of our simulation-based likelihood approach is well exemplified by its handling of ascertained SNP chips, and the inference of several parameters from the SFS under complex demographic scenarios. Previous ways of handling ascertained SNP chips either consisted in removing the bias induced by the ascertainment [44] or taking it into account in the estimation procedure [39], [45]. However, these methods are usually not as general as our implementation, as they are either restricted to models including a single population [44], or to the case of the sole estimation of divergence time between two populations [45]. Contrastingly, our method can be applied to various types of demographic models including several populations, bottlenecks and migration.

Our simulation results suggest that parameters of complex models can be correctly recovered when the ascertainment consists of randomly chosen SNPs heterozygous in a single individual (Figures S8 and S9). Interestingly, we find that some parameters of unascertained populations that diverged a long time ago either with (Figure S8) or without (Figure S9) admixture can also be quite well estimated when the model is well specified. This suggests that a given ascertainment panel of the GWHO Affymetrix chip could be used to infer parameters in several related populations. It is also worth noting that our calibration of parameters relied on the assumption that the divergence time with an outgroup population was known, but a different divergence time would only require a rescaling of the estimated parameters. The use of an outgroup species with fixed divergence time is a standard way to calibrate mutation rates (as e.g. in [21]), but we note it could also be used within species for DNA sequence data when some uncertainty exist on mutation rates, which is currently the case in humans [67], [68].

Most parameters inferred from real African populations have very similar estimates and confidence intervals irrespective of which SNP panel is used (Figure 5, Table 2), which agrees with our simulation results (Figures S8, S9). However, a few parameters seem to provide relatively divergent estimates, like the Yoruba and the African ancestral size, as well as the Yoruba-San divergence time, a discrepancy that is not really expected from the simulations. This discrepancy could stem from either an unknown source of ascertainment, from a misspecification of the model for one of the two ascertained population, or from an ascertained individual that is not representative of its population, the latter case being possibly due to inbreeding or admixture. It currently appears difficult to disentangle these cases, and the inclusion of additional parameters in model B only seems to marginally improve the fit of the expected SFS to the data. It suggests that our models still do not capture all aspect of the true demography of these populations, which might also affect our ability to reproduce the ascertained SFS, and have a negative impact on our estimations. We note however that previous estimates of African demography [e.g. 53] are more in line with those inferred from the Yoruba than from the San panel, which could suggest that our demographic models are more appropriate for the Yoruba than for the San population. Overall, our results nevertheless show that meaningful demographic estimates can be obtained from ascertained SNP chips, suggesting a useful and cheap alternative to large scale sequencing for demographic inference.

### Application to complex demographic models

Our methodology has the potential to infer demographic parameters from large scale genomic data under a much wider range of neutral evolutionary models than either the current implementation of 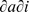, current Approximate Bayesian Computation (ABC) implementations [69], summary statistics based approaches [11], or other existing likelihood-based methods [22]. Whereas ABC has the potential to be applied to genomic data, it has rarely been done since it usually requires the simulations of data sets as large as those analysed, which is computationally very costly. Our approach could thus be seen as a powerful likelihood-based alternative to the study of complex evolutionary models, which are usually only tackled by ABC approaches [see e.g. 16], [70], [71], with the additional advantage of not having to choose which summary statistics to use for the inference, which is often a problem in ABC [e.g. 13], [72], [73], [74]. Our approach can indeed tackle complex evolutionary models with a relatively large number of populations (see Figs. 1D, 4B and 5B). For instance, the model shown in Figure 4B includes 4 sampled populations, as well as four other unsampled populations, whose demography also needs to be reconstructed. AIC analysis reveals that the cost associated to increasing model complexity is rewarded by a much better fit to the data. One should however make a distinction between the inclusion of additional parameters for a given number of populations (e.g. adding the possibility to have gene flow between populations), and the inclusion of additional populations. The addition of unsampled or ghost populations can not only modify parameter estimations but also alter our interpretation of the results (see e.g. [75], [76]). For instance, the inclusion of continents from which sampled populations received migrants (which is an attempt at taking into account the spatial structure of African populations) in Figure 4B improved the fit of expected SFS (see Table S2), without really modifying our estimation of the level of European admixture, but it radically changed our interpretation of the relationships between African Americans and extant African and European populations. As expected, the inclusion of a potential source of admixture for the Luhya population in model B2 improved the fit of the model and it allowed us to make inference about this ghost population, but it also modified estimated parameter values of this and other populations. These observations suggest that complex models are better studied by considering all populations simultaneously, and that a strategy consisting in estimating population-specific parameters and fixing them when incorporating additional populations would not be optimal.

There are still some limits to the complexity of models that can be studied, and AIC-like approaches can be used to study which modifications sufficiently improve the model to be preserved. However, the question of whether our best model is the true model is not addressed by model comparisons such as likelihood ratios or AIC. One would ideally like to assess how well the model explains the data, which is usually done by some posterior predictive check in a Bayesian setting [77], or by getting the data p-value under a frequentist approach. We have implemented such an approach, where the model p-value was evaluated by comparing an observed G-test statistic [3], [62] to its model distribution. As expected, this approach leads to non-significant p-values when applied to simulated data sets (Figure S11). However, the p-values for all models shown in Figures 4 and 5 are highly significant (*p* = 0, Figures S12 and S13) suggesting that our implemented models of human evolution are still overly simplistic. This is not surprising given the high-dimensionality of the parameter space and the large amount of SNPs at hand giving us high power to reject inaccurate hypotheses. Since models are generally expected to be wrong, the question is at what point is a model so wrong that it is no longer useful [78, p. 74]. The fact that the addition of plausible source of realism into our models significantly improves the fit to the data (Tables S2 and S3) is reassuring in the sense that we have a methodology to refine our still imperfect evolutionary scenarios.

## Methods

### Simulation-based site frequency spectrum and likelihoood

Nielsen [17] has shown that one could estimate the likelihood of a demographic model 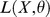, where *X* is the site frequency spectrum, on the basis of coalescent simulations. This is because the probability 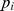 of a given derived allele frequency *i* is simply a ratio of branch lengths of the coalescent tree expected under model 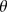 as [17]:
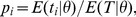
(1)where 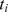 is the total length of a set 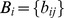 of branches directly leading to *i* terminal nodes, and *T* is the total tree length. This probability can then be estimated with arbitrary precision on the basis of Z simulations as [62]

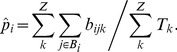
(2)where 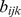 is the length of the *j*-th compatible branch in simulation *k* (see Figure S1A). Note that the estimator shown in eq. (2) implicitly weights simulations according to the probability that a mutation occurs on the simulated tree. Note that an estimator of the form 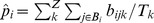 (as used by Garrigan [22] to estimate the expected SFS) would give each tree the same weight and would thus give an excessive weight to genomic regions with shallow coalescent trees, which can be a problem for recently bottlenecked populations. If some simulated entries of the SFS were zero (because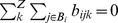), 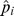 was set to an arbitrarily small values [as in 22] chosen here as 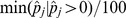.

We have empirically checked that our procedure gives the correct SFS under two simple scenarios for which the expected SFS can be obtained exactly by the method developed by Chen [18] for cases involving up to two populations and no migration. These scenarios were (i) a bottleneck model (as in Fig. 1A) and (ii) a divergence model without migration (as in Fig. 1B but without migration). We show in Figures S14 and S15 for scenarios i) and ii), respectively, the fit of the SFSs entries (estimated by our approach for different numbers of coalescent simulations) to the true SFS entries. As expected the fit improves with the number of simulations, and the estimated SFS entries are distributed symmetrically around the true values without any visible bias for these two scenarios.

### Composite likelihoods

Probabilities inferred from the simulations and eq. (2) can then be used to compute the composite likelihood of a given model as [20]

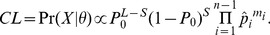
(3)where 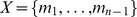 is the SFS in a single population sample of size *n*, *S* is the number of polymorphic sites, *L* is the length of the studied sequence, and 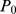 is the probability of no mutation on the tree, obtained as 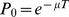 assuming a Poisson distribution of mutations occurring at rate 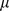.

This formulation can be extended for the joint SFS of two populations as
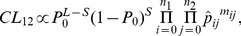
(4)and one can define a *v*-dimensional SFS for more than two (*v*) populations as
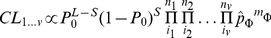
(5)where 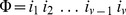 is a composite index. However, when the number of populations in the model is larger than 2 and sample sizes are relatively large, the number of entries in the v-dimensional SFS can be huge, implying that most entries of the observed SFS will be either zero or a very small number and that the expected values for these low-count entries will be difficult to estimate precisely. In that case, we have chosen to estimate the *v*-dimensional 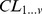 by collapsing all entries with observed SFS less than a predefined threshold 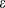 as
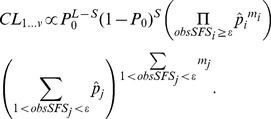
(6)When *v*>4, this approach will also prove computationally difficult, and in that case we have chosen to compute a composite composite-likelihood (*C2L*) obtained by multiplying all pairwise *CL*'s, as
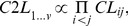
(7)where 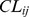 is given by eq. (4).

### Maximizing the likelihood

As the likelihood is obtained by simulations, which incurs some approximation, we cannot use optimization methods based on partial derivatives. Even though other methods would be possible, we have chosen to use a conditional maximization algorithm [ECM, 79], which is an extension of the EM algorithm where each parameter of the model is maximized in turn, keeping the other parameters at their last estimated value. The maximization of each parameter was done using Brent's [80, Chapter 5] algorithm, which is a root-finding algorithm using a combination of bisection, secant and inverse quadratic interpolation [see e.g. 81]. We start with initial random parameter values, and perform a series of ECM optimization cycles until estimated values stabilize or until we have reached a specified maximum number of ECM cycles (usually 20–40). Unless specified otherwise, we used 100,000 coalescent simulations for the estimation of the expected SFS and likelihood for a given set of demographic parameters. Even though a higher precision could be reached with a larger number of simulations, especially for complex models, this number appears like a good compromise between computational efficiency and likelihood estimation accuracy (see Figure S2). Note that the imprecision on the likelihood estimation might also prevent an efficient optimization of our parameters, as a sub-optimal parameter might give by chance a better likelihood than the optimal one during an ECM cycle. Because the composite likelihood surface might have several local maxima and be difficult to explore [e.g. 60], several independent optimizations are performed (between 20 and 40 depending on the model and computation time), each starting from different initial conditions, and the overall maximum composite likelihood solution is retained.

Coalescent simulations, estimation of the SFS, likelihood computations and its maximization were all done with *fastsimcoal2*, a modified version of the *fastsimcoal* program [82]. *fastsimcoal2* input file format and command lines arguments are briefly described in Supplementary Text S1, and examples of input files are provided in Supplementary Text S2.

### Tested demographic models without ascertainment

We have tested our program ability to recover demographic parameters from DNA sequence data in four relatively plausible but distinct scenarios of population differentiation involving one to ten populations with migration (see Figure 1). In all cases, we simulated with *fastsimcoal2* 400,000 unlinked regions of 50 bp, thus totaling 20 Mb of DNA sequences, assuming a mutation rate of 2.5×10^−8^ bp^−1^ per generation and an infinite-site model. Pseudo-observed SFS were also directly computed with *fastsimcoal2*. Parameters were estimated independently from ten data sets generated under each model. For each data set generated under models with one to three populations, we performed 50 parameter estimations via ECM maximization, and each time retained the parameter set with maximum likelihood. For the model with 10 populations we only performed 20 estimations per data sets, and used 50,000 simulations instead of 100,000 for the other models to estimate the expected SFS due to long computation times. We describe the four tested models in Figure 1, and the used parameter values are showed as red dots in Figures 2, 3, S4 and S5. Absolute numbers (generations, population sizes) were obtained by assuming that the mutation rate of 2.5×10^−8^ bp^−1^ per generation was known.

As a benchmark, we used 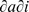 to infer the demographic parameters in scenarios shown in Figure 1A–1C involving up to three populations. For each generated data set, we performed 50 parameter estimations using the Broyden-Fletcher-Goldfarb-Shanno (BFGS) optimization method implemented in 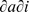, and we retained the parameters associated with the maximum likelihood. We followed 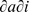
's manual specification to set reasonable upper and lower bounds of the search ranges of the parameter. In all cases, the expected SFS was estimated by extrapolating the SFS inferred from 3 grid sizes set to 40, 50 and 60, which are in all cases larger than our maximum samples sizes (30 in the IM model case). The composite likelihood was computed using 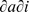
's multinomial model, which is in fact a product of Poisson likelihoods, where the expected model entries are scaled to sum up to 1. This likelihood also ignores information about the expected and observed numbers of monomorphic and polymorphic sites used in our likelihood formulation (as well as in [20]). Therefore, the ratio 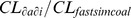 should be equal to 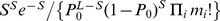 showing that barring the 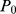 terms, the two *CL*s differ by a single constant value. The difference between likelihoods computed with fastsimcoal and 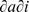 is illustrated in Figure S3 for the case of the bottleneck scenarios shown in Figures 1A. It shows that when monomorphic sites are not taken into account, *fastsimcoal* and 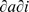 indeed produce essentially identical likelihood profiles around true parameters. However, when monomorphic sites are used in the likelihood, the shape of the likelihood profiles differs, making it more or less peaky depending on the parameter. There is thus no clear advantage in using one or the other likelihood form for this scenario, but our use of monomorphic sites allows us to directly get absolute values of the parameters. We report in Figures 2, S4 and S5 only the results obtained for data sets for which 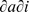
's best log likelihood was less than 10% lower than the largest log-likelihood obtained with the other data sets, and we considered 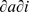 not to have converged for the discarded data sets.

### Estimating demographic parameters from an ascertained SNP array

Recently, Affymetrix developed a new SNP array including ∼629,000 SNPs with known ascertainment scheme for population inference (Axiom Genome-Wide Human Origins 1 Array, http://www.affymetrix.com/support/technical/byproduct.affx?product=Axiom_GW_HuOrigin) [43]. This array, abbreviated hereafter GWHO, is made up of SNPs defined in 13 discovery panels. In the first 12 panels, SNPs have been identified by comparing the two chromosomes of an individual from a known population, further quality checks and validation on a large population sample [43]. The 13th panel contains SNPs that are polymorphic when comparing the Denisovan sequence and a random San chromosome. Raw genotypes from 943 unrelated individuals from more than 50 worldwide populations are freely available on ftp://ftp.cephb.fr/hgdp_supp10/.

The ascertainment scheme of this array is simple and homogeneous over a given panel. However, the SFS inferred from this array is biased as only mutations that occur in the ancestry of the two compared chromosomes will be considered (see Figure S1B). We show in Figure S7 the difference between the ascertained and non-ascertained SFS under a few basic demographic scenarios in a single population. The differences between the two SFS can be quite dramatic, implying that the estimation of demographic parameters on ascertained data sets without taking the ascertainment into account is bound to lead to biased estimates. Nielsen et al. [44] have shown how to correct the expected SFS within a given population under such a simple ascertainment scheme, and the ascertained joint SFS could be unbiased in a similar way by taking into account ascertainment probabilities in the ascertained populations. Rather than unbiasing the SFS, we have chosen here to incorporate the bias in the model and to infer demographic parameters directly from the ascertained (joint) SFS, a strategy similar in spirit to that used by Gravel et al. [28] to account for biases in the SFS obtained from low-coverage next-generation sequencing data. It implies we need to model the ascertainment scheme in the coalescent simulations such as to infer the expected ascertained SFS for a given demography. In order to estimate the SFS when SNPs are defined as being sites heterozygous in a given individual, we use the following procedure: 1) we perform conventional coalescent simulations under a given demography, 2) we choose two lineages at random in the ascertained population, 3) we identify the subtree relating the chosen lineages to their most recent common ancestor (MRCA) (highlighted in blue in Figure S1B), 4) we update the numerator in eq. (2) by summing up branch lengths of the blue subtree that are ancestral to *i*_1_ lineages in population 1, *i*_2_ lineages in population 2, …, *i*_v_ lineages in population *v*, 5) The denominator of eq. (2) is updated by summing up the total length of the blue subtree.

Parameter optimization is then performed similarly to the unascertained case, except that the terms depending on the number of monomorphic sites (
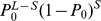
) in eq. (6) are removed from the likelihood since only polymorphic sites are reported on the ascertained chip, which implies that we cannot use a molecular clock. Therefore, parameter absolute estimation should be done relative to an arbitrarily fixed or known parameter (e.g. population size, divergence time). Note however that a molecular clock could be used if the fraction of sites found heterozygous were known in ascertained individuals, as in this case the expected fraction of monomorphic sites would then simply be 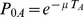, where 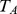 would be the total length of the expected ascertained tree (shown in blue in Figure S1B).

### Applications to human demography

#### Non-ascertained human genomic data set

We illustrate the potential of our method to inferring demographic parameters from more than three populations by investigating the past demography of four populations analyzed by Complete Genomics, which sequenced the whole genome of 54 unrelated individuals from 11 populations at a depth of 51-89X per genome [83]. This data set was chosen as we could assume that heterozygous positions were recovered unambiguously due to the high coverage, and because it covered non-genic regions that are less sensitive to selection, and thus to bias in the site frequency spectrum [5], [84]. We thus considered autosomal SNPs found outside genic regions (as defined by Ensembl version 71, April 2013 [85], and outside CpG islands (as defined on the UCSC platform, [86]). The derived and ancestral states of the SNPs were inferred from the comparison with the chimpanzee and orangutan genomes, using the syntenic net alignments between hg19 and panTro2, and between hg19 and ponAbe2, both available on the UCSC platform [86]. We then kept the SNPs found to be polymorphic in 27 individuals from 4 samples representing African Americans (5 African Americans from Southwest United States), 2 African populations (4 Luhya from Webuye, Kenya; 9 Yoruba from Ibadan, Nigeria) and a European population (9 Utah residents with Northern and Western European ancestry from the CEPH collection). The multidimensional SFS for these four populations was inferred from a total of 239,120 independent SNPs located more than 5 Kb apart from each other, to minimize linkage disequilibrium.

We then considered several demographic scenarios accounting for the pattern of genomic diversity found in these samples (see Figure 4). The first investigated scenario A is shown in Figure 4A. It is a relatively simple scenario, with 12 parameters, which assumes a divergence between the European and an ancestral African population to have occurred 50 Kya [28], [48], and this date is used to calibrate the other parameters. The two Niger-Congo (NC) speaking populations (YRI and LWK) are assumed to have diverged T_NC_ generations ago, and each NC population has a different constant effective size since then. The two African populations are then assumed to have contributed to the founding of the African-American population (ASW) 16 generations ago (around 1800, assuming 25 years per generation). The African population is also considered to have gone through a bottleneck T_BOT_ generations ago with a reduced population size N_BOT_ for 100 generations (as it is the bottleneck intensity N_BOT_/100 and not its duration that conditions genetic diversity).

We have also considered two alternative and more realistic scenarios of human evolution, but requiring more parameters to estimate (16 and 19 for models B1 and B2 in Figure 4, respectively) and additional unsampled populations (3 and 4, respectively). The main difference with the previous model A is that we assume that the European and the two NC samples belong to two different subdivided populations modeled here as continent-island systems. These continent-island models are equivalent to infinite-island models [e.g. 87], and have been shown to well approximate patterns of diversity after range expansions [47]. In this model, the CEU population receives migrants from a European continent, which is also the source of admixture of the African-American ASW population. The ASW population has also received an initial genetic contribution 16 generations ago from a Niger-Congo “continent”, which is also the source of gene flow to the two NC populations. These two continents are assumed to have diverged some time ago from an African population, and here again this time is fixed to 2000 generations for the European continent, whereas this time is estimated for the NC continent. For simplicity, all islands are assumed to have been formed 100 generations ago, which is thus the assumed duration of the scattering phase *sensu* Wakeley [88]. It implies that in the backward coalescent process, all genes remaining in the islands are transferred to the continents 100 generations ago. Note that we also allow for bottlenecks of size (N_BNC_ and N_BEUR_) to have occurred for 100 generations in the NC and European continents, respectively. Like in the model shown in Figure 4A, we allow for a different population size (N_ANC_) of the ancestral population some T_BOT_ generations ago. In our most complex model B2, we include a potential admixture of the Luhya population with an unsampled (potentially East African) population, which contributed with a proportion a_EA_ to the initial Luhya population 100 generations ago. In the simpler model B1, the Luhya population is not considered as admixed and only receives genes from the NC continent, and this model thus has 3 parameters less (16) than our model B1 (19).

Parameter estimations were performed from the multidimensional SFS, without considering the number of monomorphic sites, which were not available in the VCF files from Complete Genomics. Fixing African-European divergence time and African-American admixture times nevertheless allowed us to get absolute values for the other parameters. In addition to ignoring terms in 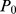 from equation (6) (command line option –0 of fastsimcoal2), we also collapsed all SFS entries less than 5 in a single category (command line option –C5 of fastsimcoal2). Maximum CL parameters were obtained after 40 cycles of the ECM algorithm, starting with 50,000 coalescent simulations per likelihood estimations, and ending with 250,000 simulations per likelihood estimation.

#### SNP chip data sets with known ascertainment

We applied our approach to infer the demographic history from ascertained SNP chips to the case of the divergence between two African populations, the Yoruba from Ibadan (Nigeria) and a hunter-gatherer San population from Southern Africa, where one individual from each population was included in the Affymetrix ascertainment panels. We can thus use the Affymetrix panel 4 (San ascertained) and panel 5 (Yoruba ascertained) to perform separate estimations that can be compared to each other. The assumed models of African population divergence we analysed are shown in Figure 5. In the simple model shown in Figure 5A, the 12 San and 44 Yoruban individuals were assumed to be drawn from two currently large populations that expanded recently from two ancestral populations that diverged T_DIV_ generations ago. We also allowed the Yoruba and San populations to have had a single pulse of bi-directional and potentially asymmetrical gene flow. As mentioned above, the use of SNPs does not allow us to get absolute dates due to the absence of a mutation clock. We therefore included the Denisova population (and its SNP data) in our model, and assumed that it diverged 400,000 years ago [58] (or 16,000 generations assuming a 25 y generation time) to calibrate our estimates. Note that an absolute dating would have also been possible if the total number of heterozygous sites per ascertained individual had been know or made available. In model B shown in Figure 5B, we use a more complex scenario describing the divergence of two continent-island metapopulations, in a way similar to models in Figure 5B. In this case we assume that the Yoruba and San sampled populations receive 2 Nm genes per generation from their respective continents, which diverged T_DS_ generations ago. At that time the San population began an exponential growth, whereas the exponential growth of the Yoruban continent was allowed to start later, T_EY_ generations ago. Note also that in Model B, the Ancestral Yoruban population and the San population can continuously exchange migrants during the period between T_EY_ and T_DS_.

Panels 4 and 5 originally contained 163,313 and 124,125 SNPs, respectively. From this we discarded all CpG SNPs potentially target of multiple hits (N. Patterson, personal communication), as well as all SNP positions with missing data in either Denisova, Yoruba or San samples, which finally left us with 109,020 and 81,383 SNPs in panels 4 and 5, respectively. The SNP ancestral states were assumed to be those present in the chimpanzee outgroup, as provided in the HGDP data set. With this choice, it is possible that some ancestral states are mis-specified (due to recurrent mutations or incomplete lineage sorting in both humans and chimps). Such misspecifications can artificially increase the observation of high-frequency derived allele classes, a signal that can wrongly be taken as the effect of selection [63]. However, this problem is relatively unlikely here, as such mis-identification should be more frequent at CpG SNPs, which were removed from our data set. Moreover, the observed SFS for both panels do not show a particular excess of high frequency derived alleles (see Figure S10). Parameter confidence intervals were obtained 100 parametric bootstrap, simulating SFS with the same number of SNPs from the CML estimates and re-estimating parameters each time. Parameters were estimated from 30 starting conditions for each original data set as well as for the 100 bootstrapped SFS data sets. Parameters associated with the maximum CL computed from eq. (6) without terms in 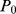 were retained after 20 cycles of the ECM algorithm were retained. One hundred thousand coalescent simulations were used to estimate the expected joint ascertained SFSs, and a threshold *ε* value of 2 was used for pooling rarely observed SFS entries in a single category, as in eq. (6) above.

#### Assessing the accuracy of estimations from ascertained SNP panels

In order to see if the 12 parameters of model A could be correctly recovered by our approach, we simulated 10 data sets under the model shown in Figure 5A using fixed parameter values, as reported in Figure S8. We simulated 20 Mb of DNA sequence data under the selected scenario and retained all SNPs that were heterozygous in an arbitrary individual from the ascertained population (pseudo-San or pseudo-Yoruba), adjusting the mutation rate to get approximately 100,000 SNPs to match the observed data. We also simulated ascertained SNP data sets under a slightly simpler scenario, removing any admixture event between Yoruba and San ancestors, as shown in Figure S9.

Parameters were estimated for the 10 data sets generated for each panel and results were reported in Figures S8 and S9.

### Model comparisons

As mentioned in the next section on model test, it might be difficult to accept a simple model with a G-test based on tens of thousands of polymorphic sites, but in that case, it might be better to establish a procedure allowing one to improve models, by progressively adding some realism to simple models [89]. Our likelihood-based approach would in principle lend itself to model comparison through likelihood-ratios for nested models or through Akaike Information Criterion (AIC, [52]) for other model comparisons. However, we are here confronted with two distinct problems. The first one affects all composite likelihood approaches and due to the fact that the distribution of the composite likelihood ratio test (CLRT) is generally unknown. When the SFS is obtained from DNA sequences with relatively large levels of linkage disequilibrium, it has been proposed to obtain an empirical distribution of the CLRT by simulation of DNA sequences with recombination (e.g. [54], [90]). In the case of the *AIC*, Varin and Vidoni [91] have proposed to replace the number of parameter *d* of a given model in 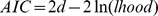 by an effective number of parameters 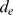 that needs to be computed from a sensitivity matrix and Godambe Information matrix, which might be difficult to do in practice. We note however that in our two applied examples the SFS is computed from a collection of SNPs randomly distributed across the genome, such that we shall conservatively assume that the CL computed from the multidimensional SFS is close to a true likelihood. Note that this assumption would not be valid if one had computed a composite likelihood based on the product of pairwise composite likelihoods, like in eq. (7). The second problem is linked to the fact that we estimate the likelihood with some error (Figure S2). As noted previously, this can prevent us to efficiently optimize our parameters, but it also means that the likelihood ratios or AIC statistics are imprecisely estimated. To address this problem, we have compared models on the basis of the maximum value of the likelihood obtained over 100 estimations performed for the ML parameters obtained by our optimization procedure. We then calculated the relative likelihood or the Akaike's weight of evidence in favor of the *i*-th model as (see e.g. [89])
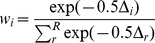
where 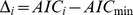.

### Model test

Even though we can estimate parameters under any model, it can be useful to check how data fit the chosen model. To this aim we use an approach based on a likelihood ratio G-statistic [3], [62] of the form 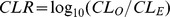, where *CL_0_* is the observed maximum composite likelihood where the expected SFS is replaced by the relative observed SFS in eqs. 3, 5 or 7, and *CL_E_* is the estimated composite maximum likelihood computed using the procedure described above. We obtain the null distribution of the *CLR* statistic by the same parametric bootstrap procedure used to infer confidence intervals, where we generate by simulation a number of data sets using the estimated maximum-likelihood parameters of the model, and each time perform parameter estimations and estimate the CLR statistic. We can compute the p-value of the observed CLR statistic from this null distribution. Note that this type of G-test has been used before to find genomic regions under selection [3], [62].

We report in Figure S11 the null distributions of the CLR and the p-values of two data sets generated under models shown in Figure 1A and 1B. In both cases, the p-values are not significant confirming that the data sets are compatible with the tested models.

Note however that a non-significant p-value is not an absolute proof that the tested model is correct, as there could be a large number of models leading to similar SFSs, as was shown previously [27], but it is an indication that the observed SFS is well explained by the model.

However, in applied cases, we actually expect that this test leads to very significant values, since the true history of the populations is completely unspecified and our models are certainly overly simple and potentially far from reality.

## Supporting Information

Figure S1 Estimation of the SFS by a ratio of branch lengths on a coalescent tree. A) Case without ascertainment bias. All branches of a given genealogy can be used to estimate the site frequency spectrum. Here we have highlighted in red the branches contributing to the SFS 2 entry. B) In case of ascertainment, only mutations occurring along the subtree (shown in blue) connecting the ascertained alleles can be observed. Therefore only branches along the blue subtree can contribute to the ascertained SFS.
(PDF)

Figure S2 Effect of the number of coalescent simulation on the precision of the estimated likelihood for different evolutionary models. The three evolutionary models considered here are those shown in Figures 1A, 1B and 1C. For each model we plot the distribution of 100 composite log-likelihoods computed with either 10,000, 100,000, 200,000, or 1 million simulations, shown as brown, orange, blue and black lines, respectively. In each case, the distributions are centered on their median value (shown below the x-axis for the 1 million simulation case), and the x axis units express deviation from the median in log_10_ likelihood units. As expected, we gain precision with increasing number of simulations performed per likelihood simulation, but the precision also decreases with the complexity of the model for the same number of simulations. Note that for computation time issues, 100,000 simulations per likelihood estimation were used for the optimizations done in the paper.
(PDF)

Figure S3Comparison of likelihood profiles between fastsimcoal2 and 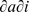 for the simple bottleneck scenario shown in Figure 1A. For each pane, we computed the likelihood of the model by varying only one parameter around its true value (blue vertical line), and keeping the other parameters of the model constant. The fourth parameter of the model, which is the ancestral population size N_A_ was explicitly set to its true value (10,000) in fastsimcoal2 and set as the reference parameter in 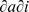, as 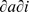 does not allow one to specify the value of the reference parameter, which might explain the small discrepancies between the blue and grey curves. The bottleneck duration was set to 100 generations for both fastsimcoal2 and 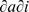. The orange and blue lines represent the scaled likelihoods computed by fastsimcoal2 with and without the monomorphic sites, respectively, whereas the grey line represents the likelihood profile obtained with 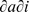. Likelihood profiles are computed around the current population size N_CUR_ = 10000 (A), the bottleneck size N_BOT_ = 100 (B), and the age of the bottleneck T_BOT_ = 1000 generations (C). One million simulations were used to compute the likelihoods of each point with fastsimcoal2. We see that when the number of monomorphic sites is not used by fastsimcoal, fastsimcoal and 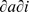 produce essentially the same likelihood up to a constant term. The use of the number of polymorphic sites makes the profile likelihood curve peakier for N_BOT_ but flatter for T_BOT_ and N_CUR_.(PDF)

Figure S4Single population bottleneck model. *fastsimcoal2* results are in black and 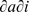
's results (9/10) are in blue. True parameters values are shown as red dots. *fastsimcoal2* required 15 minutes for a single estimation based on 40 ECM cycles over parameters, whereas 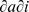 requires on average 15 seconds on a similar CPU.(PDF)

Figure S5IM model. *fastsimcoal2* results are in black and 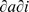
's results (8/10) are in blue. True parameters values are shown as red dots. *fastsimcoal2* required about2h30 for a single estimation based on 40 ECM cycles over parameters, whereas 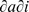 requires on average 6 minutes on a similar CPU.(PDF)

Figure S6 Marginal SFS obtained under the three models defined in Figure 4. Black lines: Observed SFS; Blue line: Fitted SFS. Gray circles: Cumulative absolute difference between observed and expected SFS.
(PDF)

Figure S7 Effect of ascertainment on the SFS in a single population. A: Stationary population. B: Population demographic expansion by a factor 100, 5000 generations ago, N_CUR_ = 50,000 diploids, N_ANC_ = 500; C: Population contraction of a factor 100, 1000 generations ago, N_CUR_ = 500 diploids, N_ANC_ = 50,000; D: Population bottleneck according to scenario shown in Figure 2A. Expected SFS were obtained from 1 million coalescent simulations. The ascertainment consisted here in selecting SNPs that were heterozygous in a single individual, like in the Affymetrix SNP array.
(PDF)

Figure S8 ML estimation of the model parameters shown in Figure 5. Parameters estimated from the simulated for the pseudo-Yoruba ascertained panel are shown in black, and the parameters estimated from the pseudo-San ascertained panels are shown in blue. For each panel, we generated 10 data sets according to parameters shown in red.
(PDF)

Figure S9 Estimation of parameters under a simpler model of population divergence between Yoruba and San. A: Demographic model and parameter definition. B: Parameters estimated from the *Yoruba* ascertained panel are in shown black, and parameters estimated from the San ascertained panels are shown in blue. True simulated parameter values are shown in red.
(PDF)

Figure S10 Observed and expected marginal SFS in San, Yoruba and Denisvova samples inferred from panel 4 and 5 for models A and B of Figure 5. Gray line = observed, dashed blue line = expected under model A of Figure 5; dashed orange line = expected under model B of Figure 5. ML estimates are reported in Table 2. Note that the fit for the expected SFS was done on the three dimensional joint SFS, and that the Denisova 1D SFS has only two entries at zero or 1.
(PDF)

Figure S11 Model tests performed for one data set generated under scenarios in Figures 1A (A) and 1B (B). The black line represents the distribution of CLRs obtained for data sets generated by parametric bootstraps from simulations done with the maximum composite likelihood parameters obtained for each data set (see Materal and Methods). The p-value of each model is then computed as the fraction of data sets with a CLR larger than or equal to the observed CLR (red line).
(PDF)

Figure S12 Model tests performed on scenartios of human history shown in Figure 4. The black line represents the distribution of CLRs obtained for data sets generated by parametric bootstraps from simulations done with the maximum composite likelihood parameters obtained for each data set and shown in Table 1. The p-values of each model are then computed as the fraction of data sets with a CLR larger than or equal to the observed CLR (red line), and are zero for all models. Note that the demographic models used for parameter estimations are clearly rejected for both SNP panels, which imply that these models cannot exactly reproduce the observed data. Note however, the shift of the red line to the right for Model B1 and B2, suggesting a much better fit to the data in line with the AIC results shown in Table S2.
(PDF)

Figure S13 Model tests performed on scenarios of African history shown in Figure 5 for the San and Yoruba SNP panels. The black line represents the distribution of CLRs obtained for data sets generated by parametric bootstraps from simulations done with the maximum composite likelihood parameters obtained for each data set and shown in Table 2. The p-values of each model are then computed as the fraction of data sets with a CLR larger than or equal to the observed CLR (red line). The demographic models used for parameter estimations are clearly rejected for both SNP panels (p-value = 0 for all models), which imply that these models cannot exactly reproduce the observed data. Note however, the shift of the red line to the right for Model B, suggesting a much better fit to the data in line with the AIC results shown in Table S3.
(PDF)

Figure S14 Fit of the SFS approximation obtained with *fastsimcoal2* to the expected SFS for a bottleneck model similar to that shown in Fig. 1A. The expected SFS was obtained by implementing eqs. 2–9 from Chen [18] in Mathematica ver 9.0.1.0. We considered a scenario with the following fixed parameter values: 2N_CUR_ = 10000, 2N_BOT_ = 100, and 2N_A_ = 10000. The sample size was fixed to *n* = 20. We examined the effect of varying the age of the bottleneck T_BOT_, as 10, 1000, and 10000 generations, which corresponds to a scaled times of 0.001, 0.1, and 1.0 in units of 2N_A_, and the effect of varying the number of simulations per SFS estimation (between 1 thousand and 1 million). A–C) Direct comparison of the SFS entries (in log scale), where we report *fastsimcoal2* results for 100 replicated runs. As can be seen, the higher the number of simulations the better the approximation, as the points get closer to the diagonal. D–F) Relative Error of the *fastsimcoal2* SFS approximation, defined as ((SFS *fastsimcoal2*-exact SFS)/exact SFS). As expected, increasing the number of simulations decreases the relative error. Interestingly, the error distributions are symmetrically distributed around zero suggesting that the *fastsimcoal2* approximation is essentially unbiased.
(PDF)

Figure S15 Fit of the SFS approximation obtained with *fastsimcoal2* to the expected SFS for a divergence model similar to that in Fig. 1B but without migration. The expected SFS was obtained by implementing eqs. 2–9 from Chen [18] in Mathematica ver 9. We considered a scenario where two populations of different sizes 2N_1_ = 10000 and 2N_2_ = 1000 diverged TDIV generations ago from and an ancestral population of size 2N_A_ = 10000. The number of sampled genes are *n*_1_ = 20 and *n*_2_ = 30 for populations 1 and 2, respectively. We examined the effect of using 1 thousand to 1 million simulations to estimate the SFS for varying TDIV values of 0.001, 0.1, and 10, expressed in units of 2N_A_ generations. A–C) Direct comparison of the SFS entries (in log scale), where we report *fastsimcoal2* results for 100 replicated runs. Note that we do not show SFS entries estimated by *fastsimcoal2* that have a value of zero, but these entries correspond to extremely low expected values. As can be seen, the higher the number of simulations the better the approximation, as the points get closer to the diagonal. D–F) Relative Error of the *fastsimcoal2* SFS approximation in log scale. The density curves were obtained after exclusion of outliers (values smaller or larger than the1% and 99% quantiles, respectively). As expected, increasing the number of simulations decreases the relative error. Interestingly, the error distributions are symmetrically distributed around zero suggesting that the *fastsimcoal2* approximation is essentially unbiased.
(PDF)

Table S1 Inferred parameters of human demography under model A of Figure 4A.
(PDF)

Table S2 Relative likelihood of the different models shown in Figure 4.
(PDF)

Table S3 Relative likelihood of the different models shown in Figure 5 for SNP chip panels 4 and 5.
(PDF)

Text S1 Input file format and *fastsimcoal2* command lines.
(PDF)

Text S2 Examples of input files used in the paper.
(PDF)
