# Supplementary figures and images for: Robust Demographic Inference from Genomic and SNP Data
Source: PLoS Genet. 2013 Oct 24;9(10):e1003905. doi: 10.1371/journal.pgen.1003905 (PMC3812088; doi:10.1371/journal.pgen.1003905)

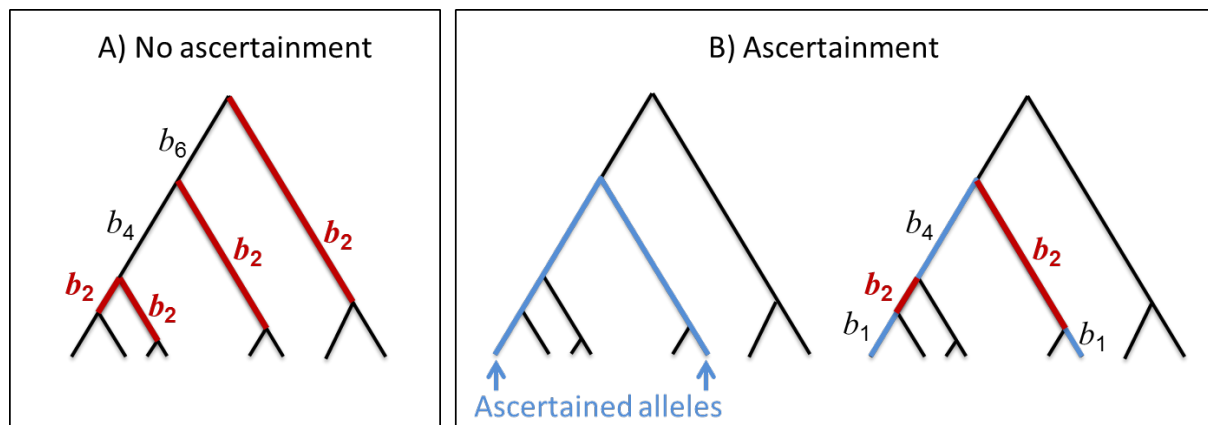

Figure S1:

Supplement: Figure S1 — Estimation of the SFS by a ratio of branch lengths on a coalescent tree. A) Case without ascertainment bias. All branches of a given genealogy can be used to estimate the site frequency spectrum. Here we have highlighted in red the branches contributing to the SFS 2 entry. B) In case of ascertainment, only mutations occurring along the subtree (shown in blue) connecting the ascertained alleles can be observed. Therefore only branches along the blue subtree can contribute to the ascertained SFS. (PDF) [file pgen.1003905.s001.pdf]

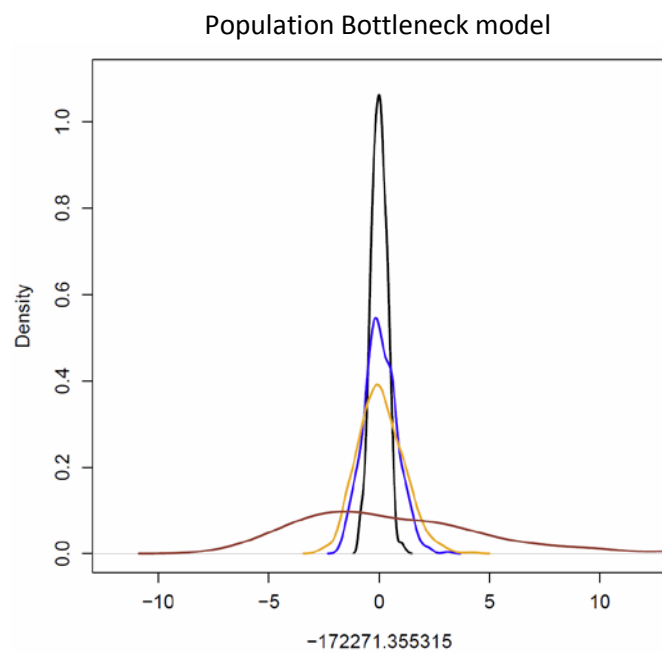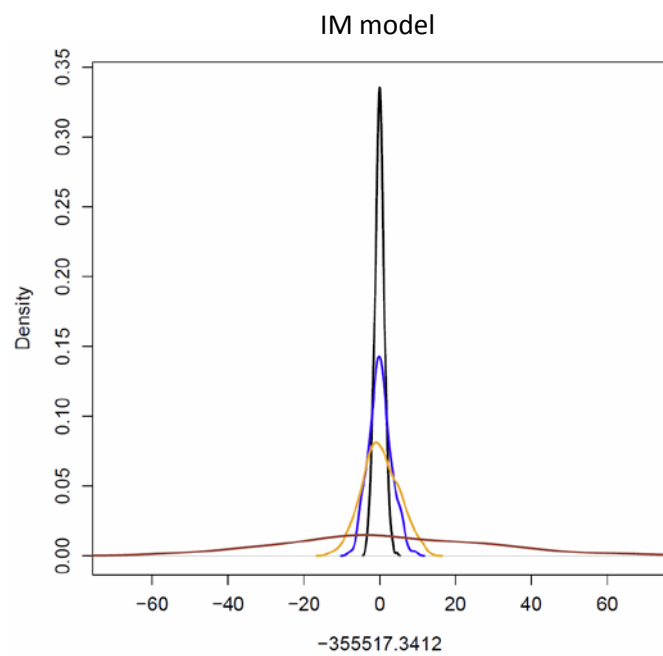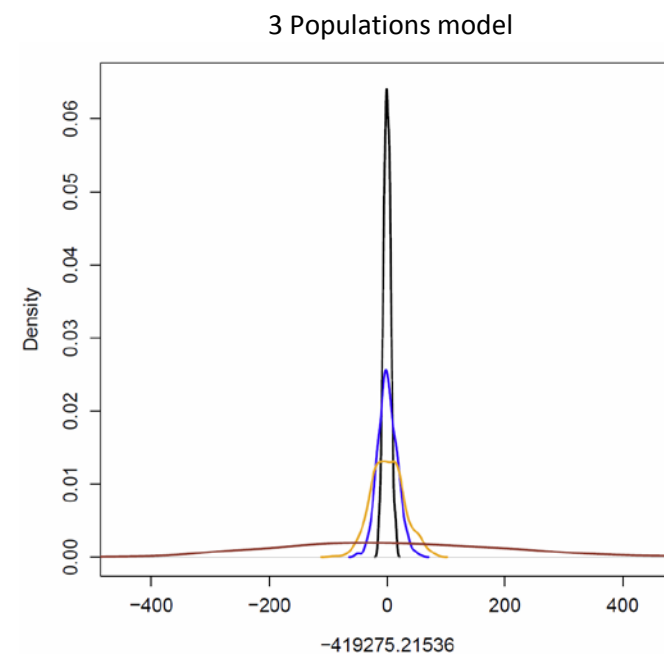

**Figure S2:**

Supplement: Figure S2 — Effect of the number of coalescent simulation on the precision of the estimated likelihood for different evolutionary models. The three evolutionary models considered here are those shown in Figures 1A, 1B and 1C. For each model we plot the distribution of 100 composite log-likelihoods computed with either 10,000, 100,000, 200,000, or 1 million simulations, shown as brown, orange, blue and black lines, respectively. In each case, the distributions are centered on their median value (shown below the x-axis for the 1 million simulation case), and the x axis units express deviation from the median in log10 likelihood units. As expected, we gain precision with increasing number of simulations performed per likelihood simulation, but the precision also decreases with the complexity of the model for the same number of simulations. Note that for computation time issues, 100,000 simulations per likelihood estimation were used for the optimizations done in the paper. (PDF) [file pgen.1003905.s002.pdf]

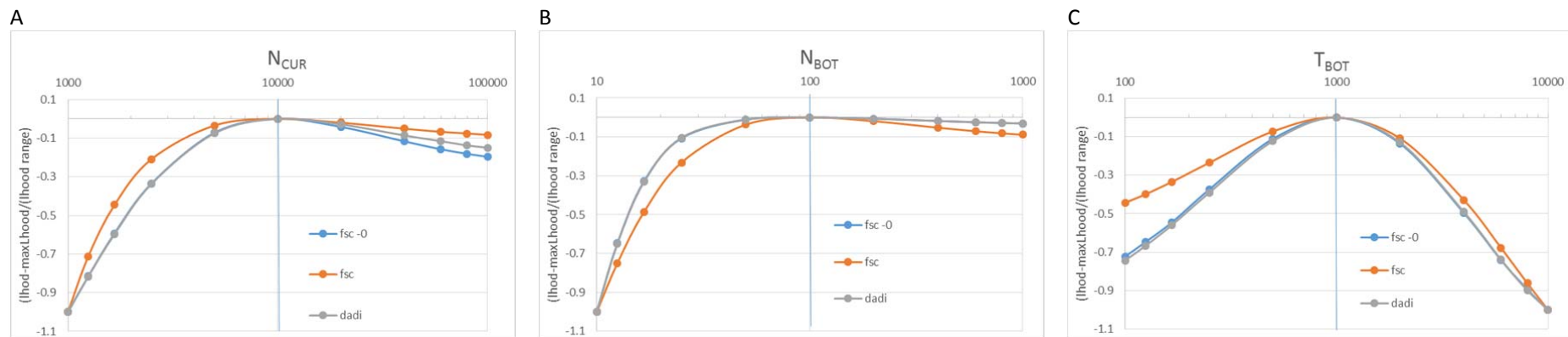

**Figure S3:**

Supplement: Figure S3 — Comparison of likelihood profiles between fastsimcoal2 and for the simple bottleneck scenario shown in Figure 1A. For each pane, we computed the likelihood of the model by varying only one parameter around its true value (blue vertical line), and keeping the other parameters of the model constant. The fourth parameter of the model, which is the ancestral population size NA was explicitly set to its true value (10,000) in fastsimcoal2 and set as the reference parameter in , as does not allow one to specify the value of the reference parameter, which might explain the small discrepancies between the blue and grey curves. The bottleneck duration was set to 100 generations for both fastsimcoal2 and . The orange and blue lines represent the scaled likelihoods computed by fastsimcoal2 with and without the monomorphic sites, respectively, whereas the grey line represents the likelihood profile obtained with . Likelihood profiles are computed around the current population size NCUR = 10000 (A), the bottleneck size NBOT = 100 (B), and the age of the bottleneck TBOT = 1000 generations (C). One million simulations were used to compute the likelihoods of each point with fastsimcoal2. We see that when the number of monomorphic sites is not used by fastsimcoal, fastsimcoal and produce essentially the same likelihood up to a constant term. The use of the number of polymorphic sites makes the profile likelihood curve peakier for NBOT but flatter for TBOT and NCUR. (PDF) [file pgen.1003905.s003.pdf]

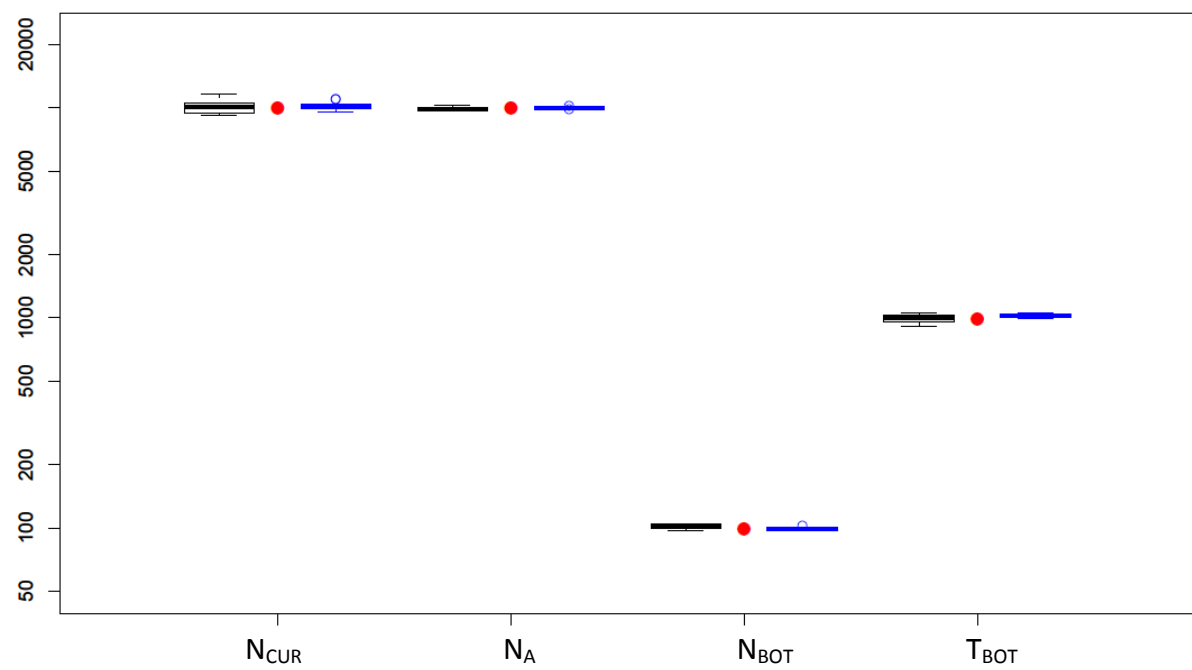

Figure S4:

Supplement: Figure S4 — Single population bottleneck model. fastsimcoal2 results are in black and 's results (9/10) are in blue. True parameters values are shown as red dots. fastsimcoal2 required 15 minutes for a single estimation based on 40 ECM cycles over parameters, whereas requires on average 15 seconds on a similar CPU. (PDF) [file pgen.1003905.s004.pdf]

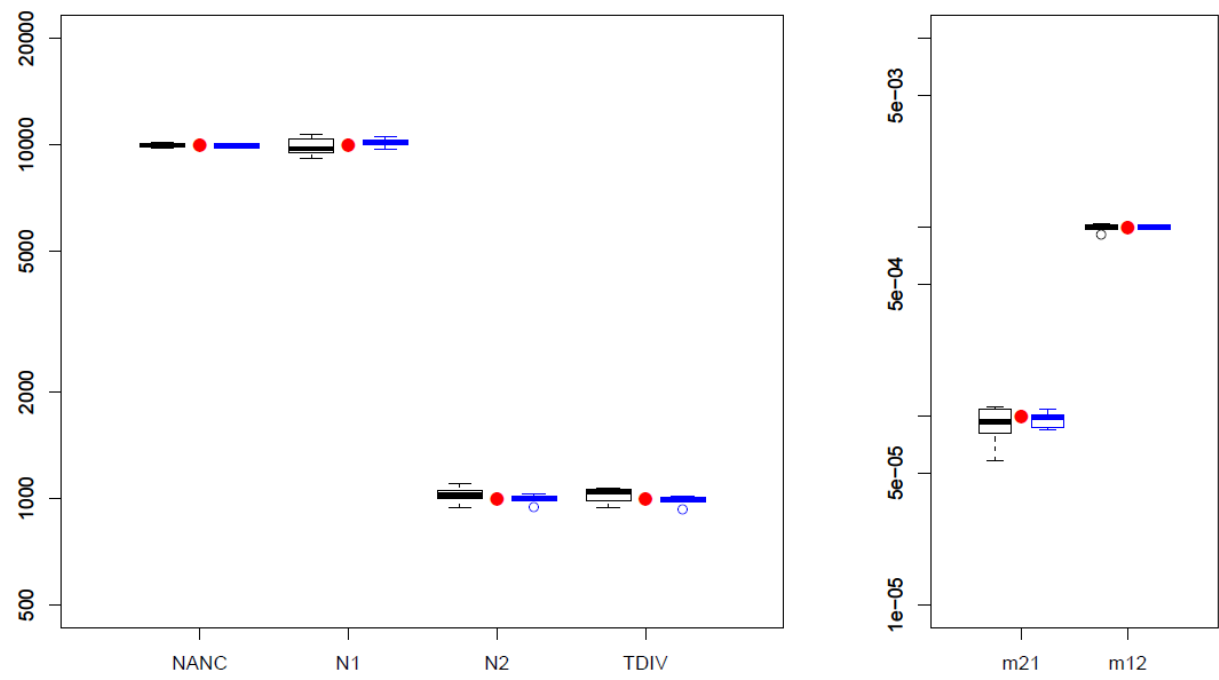

**Figure S5:**

Supplement: Figure S5 — IM model. fastsimcoal2 results are in black and 's results (8/10) are in blue. True parameters values are shown as red dots. fastsimcoal2 required about2h30 for a single estimation based on 40 ECM cycles over parameters, whereas requires on average 6 minutes on a similar CPU. (PDF) [file pgen.1003905.s005.pdf]

### Model A

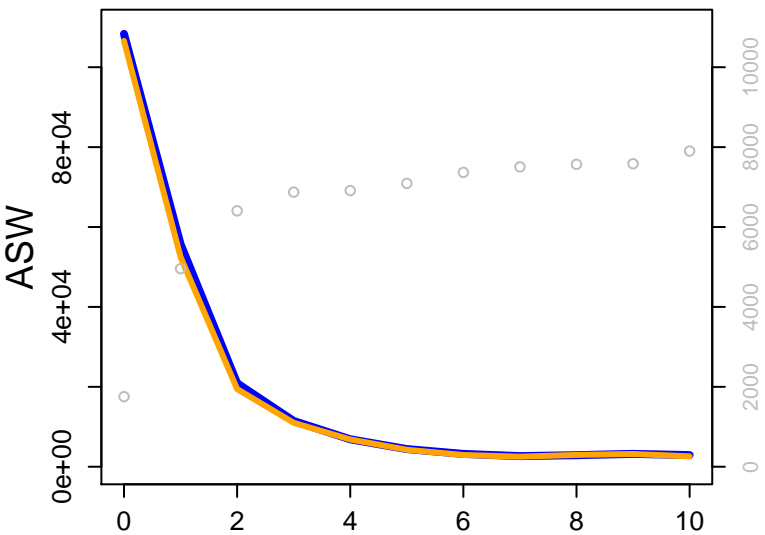

### Model B1

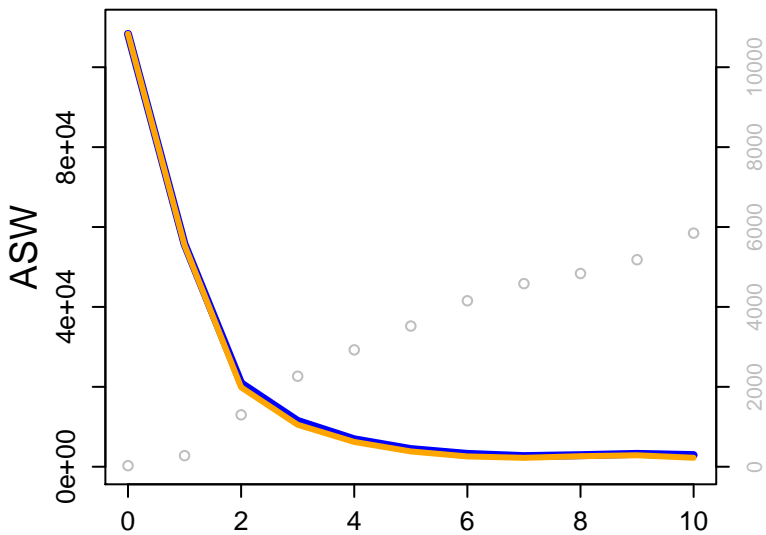

## Model B2

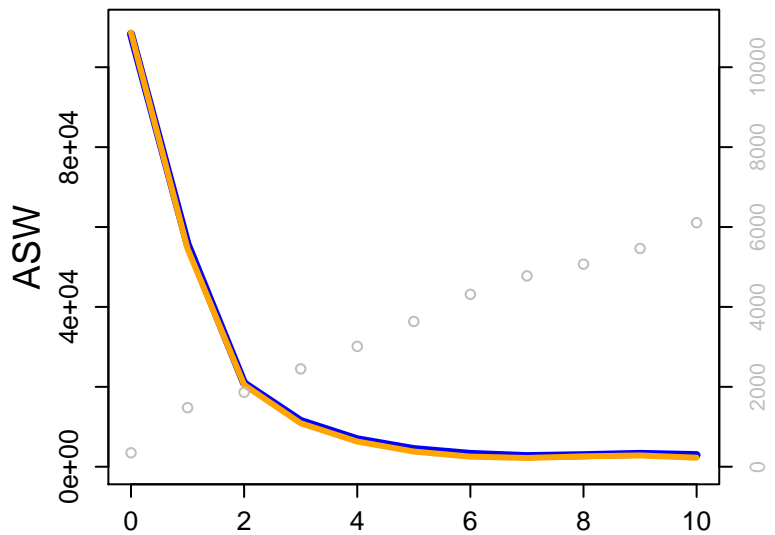

CEU

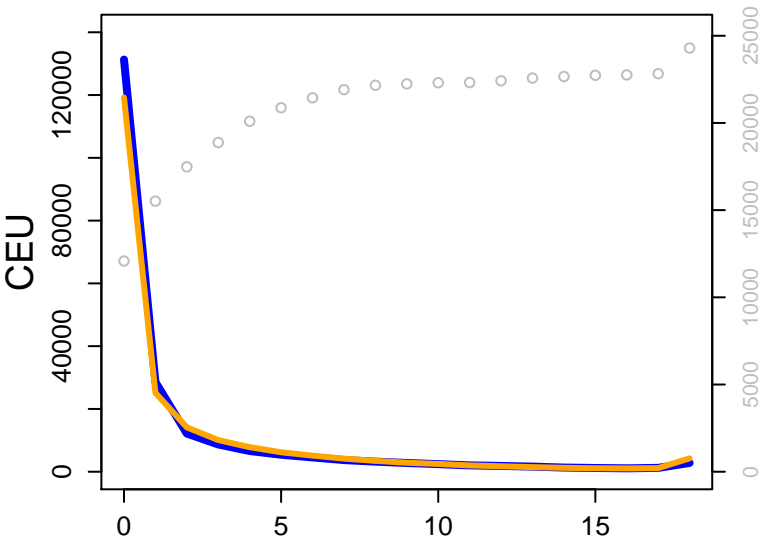

CEU

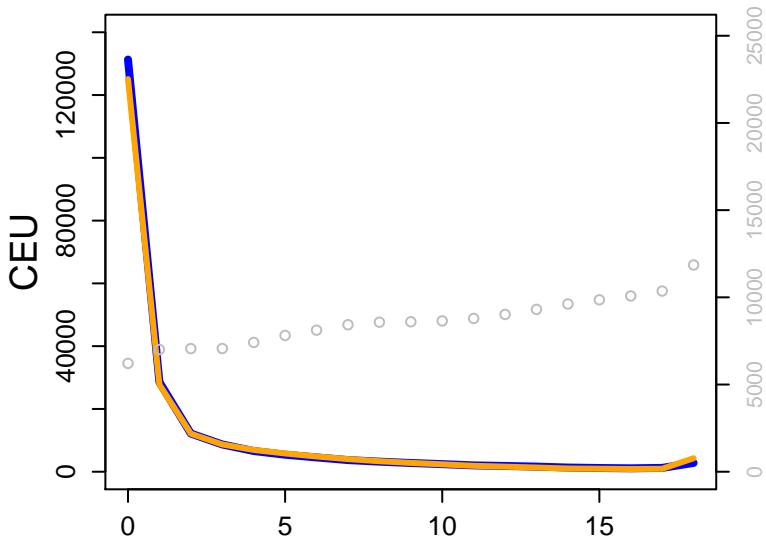

CEU

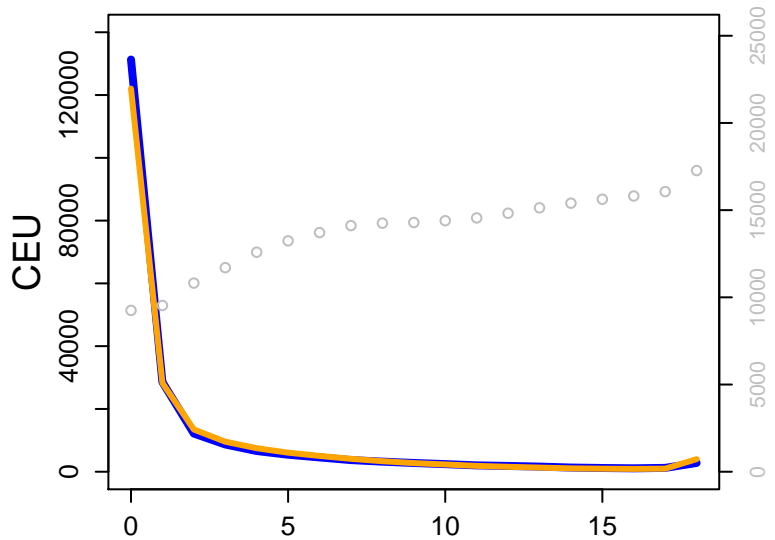

LWK

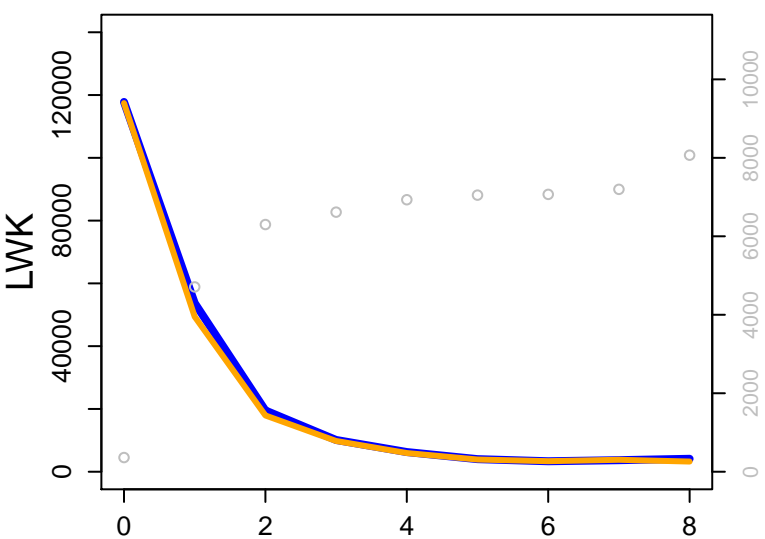

LWK

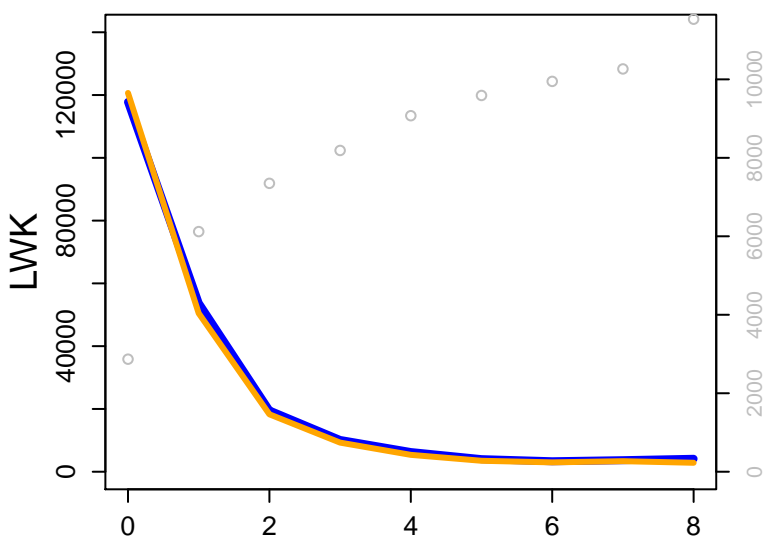

LWK

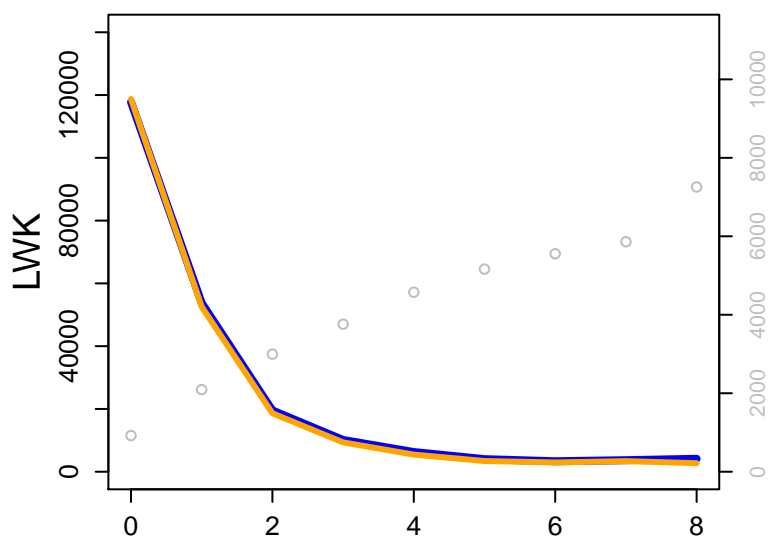

YRI

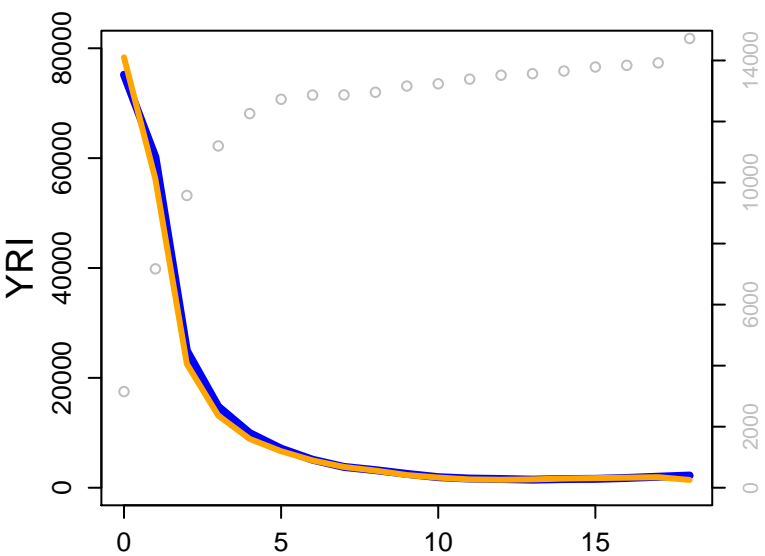

YRI

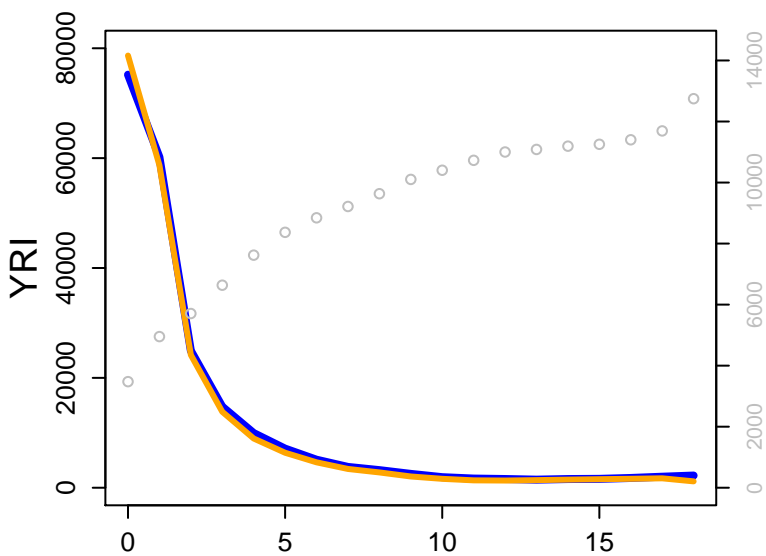

YRI

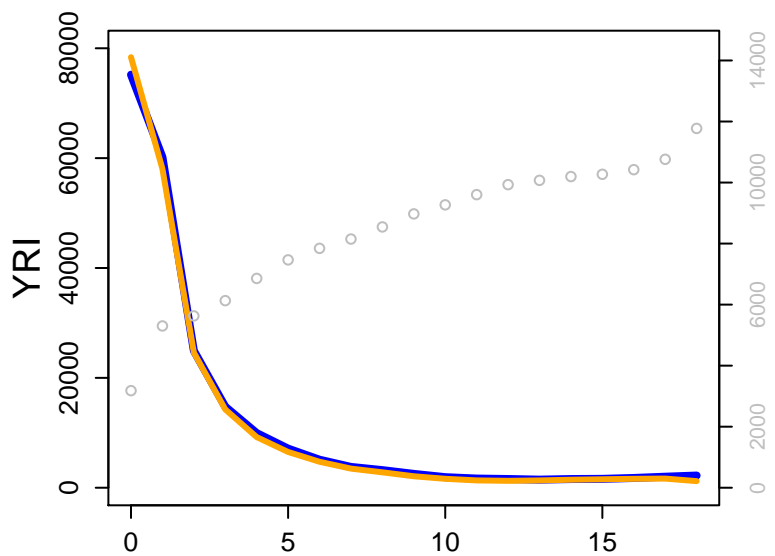

Supplement: Figure S6 — Marginal SFS obtained under the three models defined in Figure 4. Black lines: Observed SFS; Blue line: Fitted SFS. Gray circles: Cumulative absolute difference between observed and expected SFS. (PDF) [file pgen.1003905.s006.pdf]

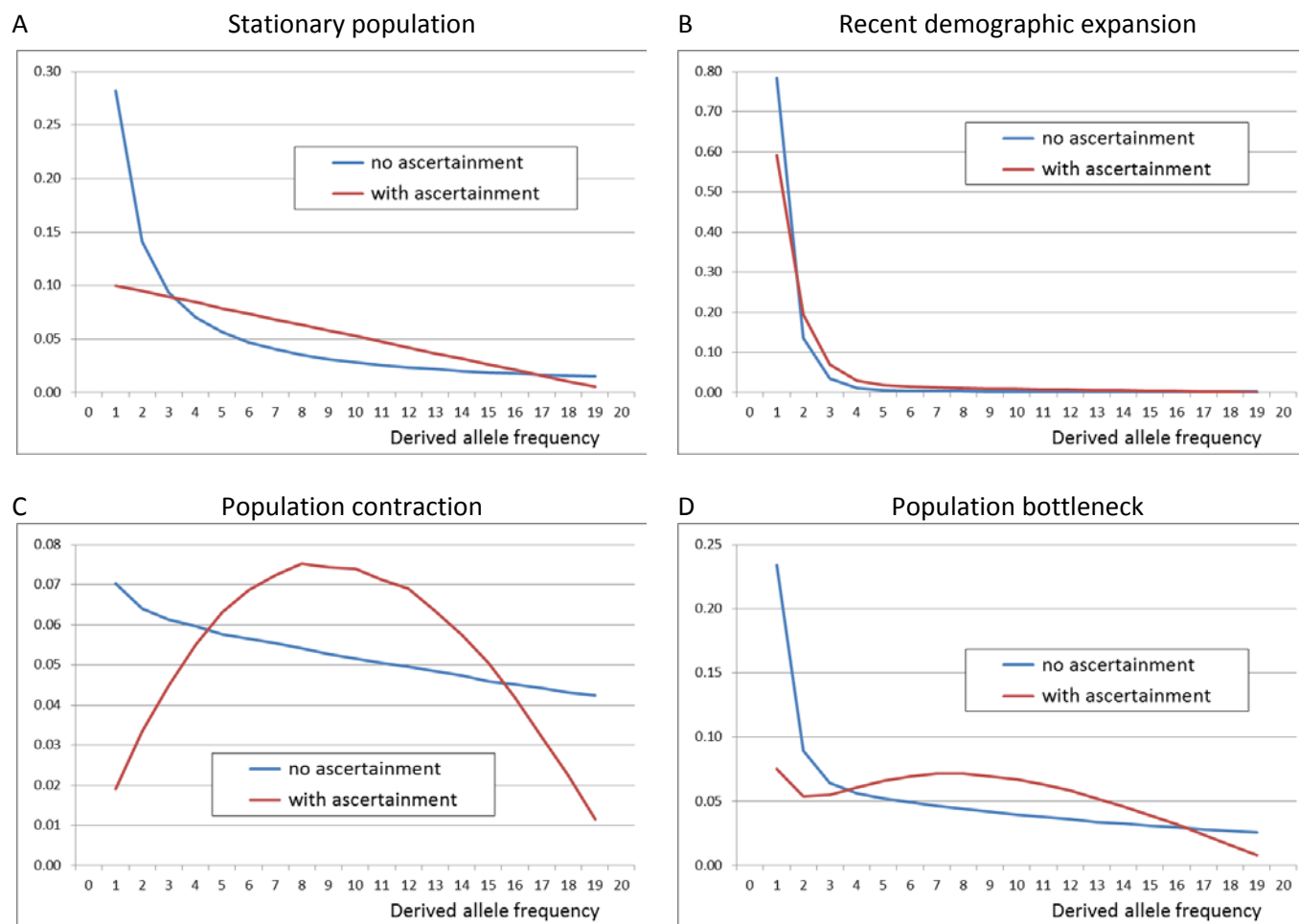

**Figure S7:**

Supplement: Figure S7 — Effect of ascertainment on the SFS in a single population. A: Stationary population. B: Population demographic expansion by a factor 100, 5000 generations ago, NCUR = 50,000 diploids, NANC = 500; C: Population contraction of a factor 100, 1000 generations ago, NCUR = 500 diploids, NANC = 50,000; D: Population bottleneck according to scenario shown in Figure 2A. Expected SFS were obtained from 1 million coalescent simulations. The ascertainment consisted here in selecting SNPs that were heterozygous in a single individual, like in the Affymetrix SNP array. (PDF) [file pgen.1003905.s007.pdf]

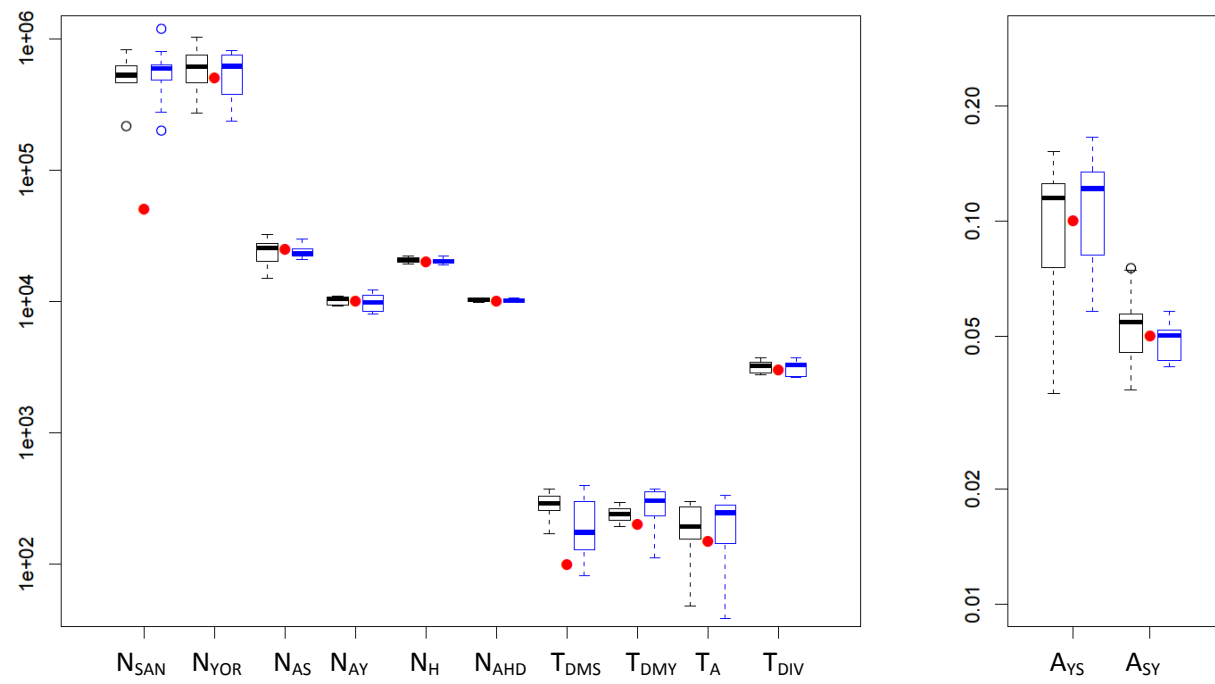

**Figure S8:**

Supplement: Figure S8 — ML estimation of the model parameters shown in Figure 5. Parameters estimated from the simulated for the pseudo-Yoruba ascertained panel are shown in black, and the parameters estimated from the pseudo-San ascertained panels are shown in blue. For each panel, we generated 10 data sets according to parameters shown in red. (PDF) [file pgen.1003905.s008.pdf]

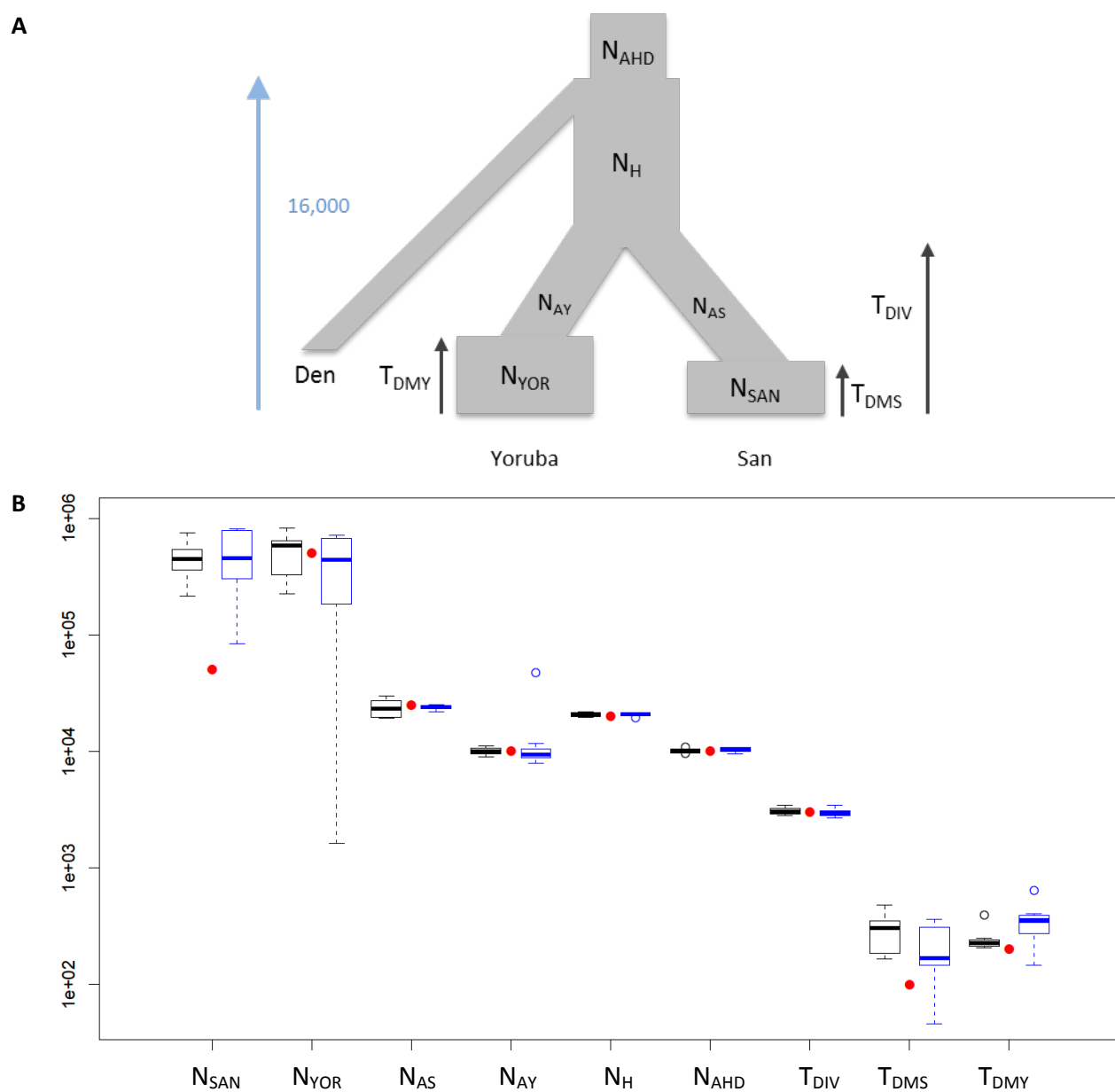

**Figure S9:**

Supplement: Figure S9 — Estimation of parameters under a simpler model of population divergence between Yoruba and San. A: Demographic model and parameter definition. B: Parameters estimated from the Yoruba ascertained panel are in shown black, and parameters estimated from the San ascertained panels are shown in blue. True simulated parameter values are shown in red. (PDF) [file pgen.1003905.s009.pdf]

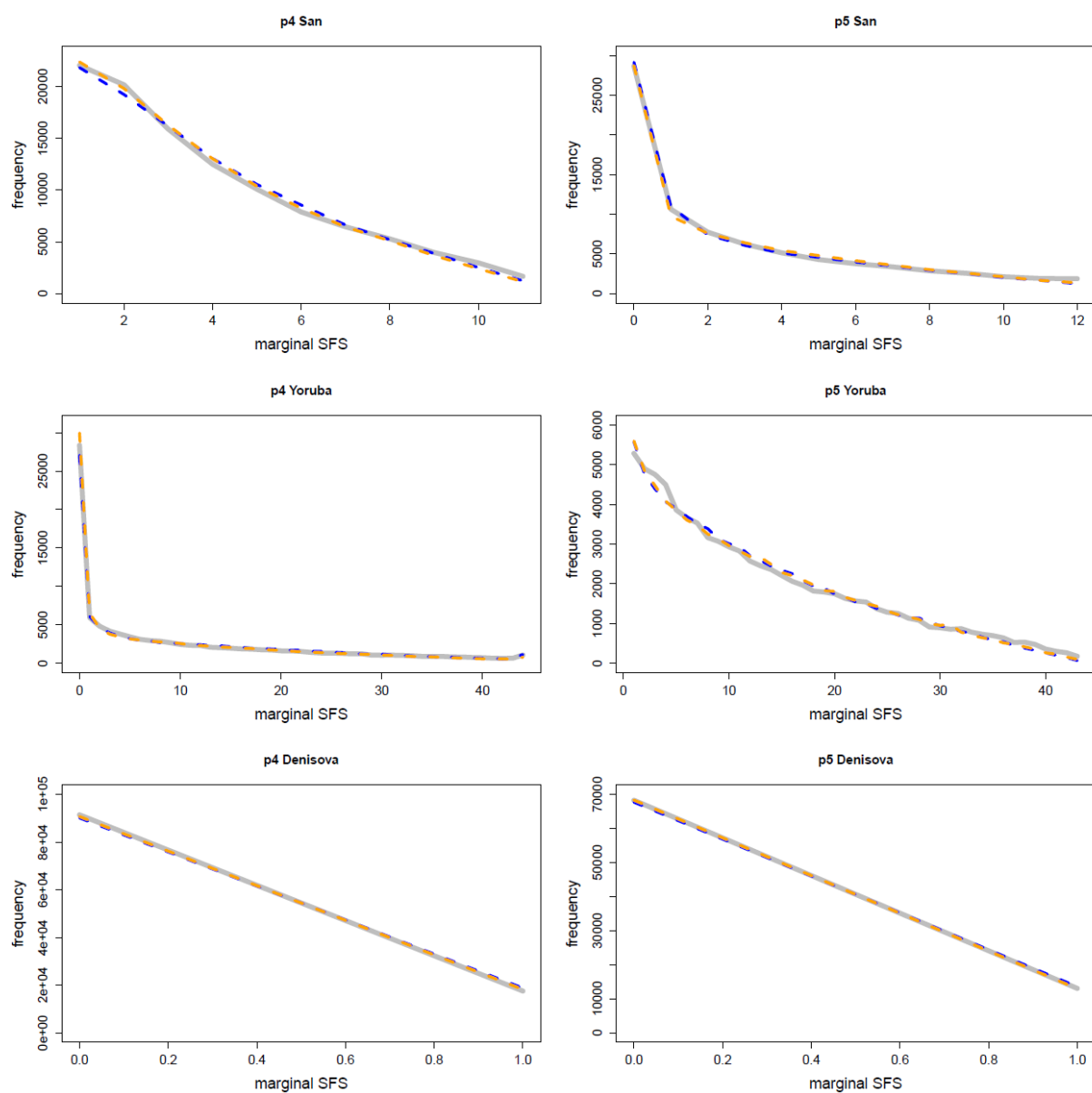

**Figure S10:**

Supplement: Figure S10 — Observed and expected marginal SFS in San, Yoruba and Denisvova samples inferred from panel 4 and 5 for models A and B of Figure 5. Gray line = observed, dashed blue line = expected under model A of Figure 5; dashed orange line = expected under model B of Figure 5. ML estimates are reported in Table 2. Note that the fit for the expected SFS was done on the three dimensional joint SFS, and that the Denisova 1D SFS has only two entries at zero or 1. (PDF) [file pgen.1003905.s010.pdf]

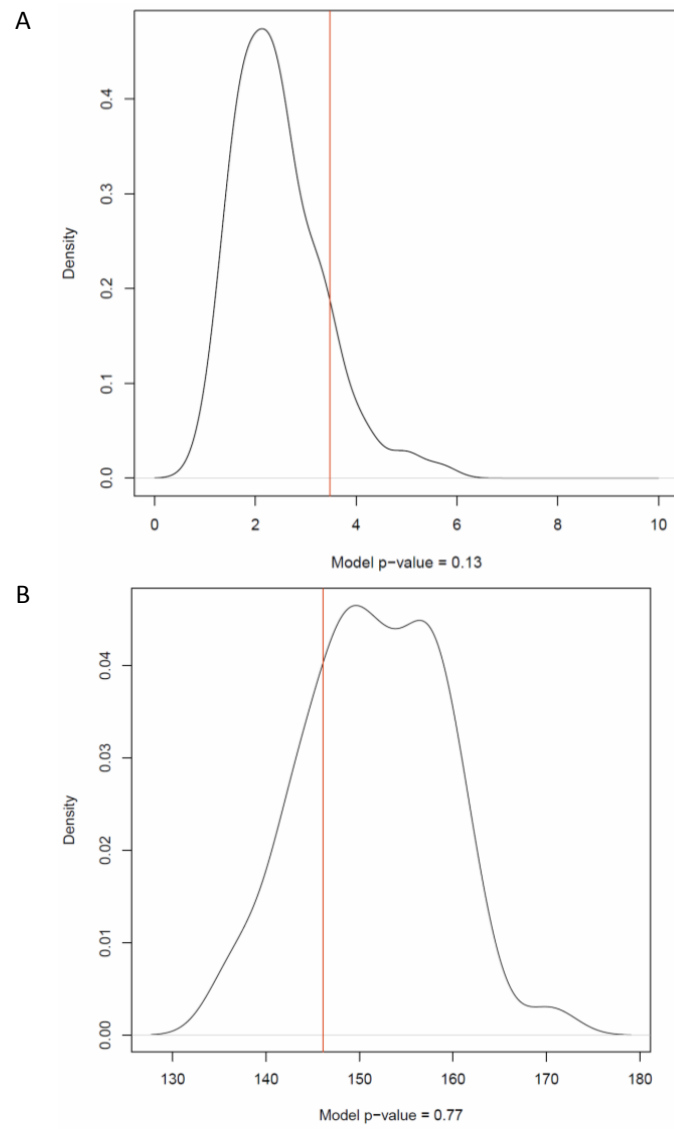

**Figure S11:**

Supplement: Figure S11 — Model tests performed for one data set generated under scenarios in Figures 1A (A) and 1B (B). The black line represents the distribution of CLRs obtained for data sets generated by parametric bootstraps from simulations done with the maximum composite likelihood parameters obtained for each data set (see Materal and Methods). The p-value of each model is then computed as the fraction of data sets with a CLR larger than or equal to the observed CLR (red line). (PDF) [file pgen.1003905.s011.pdf]

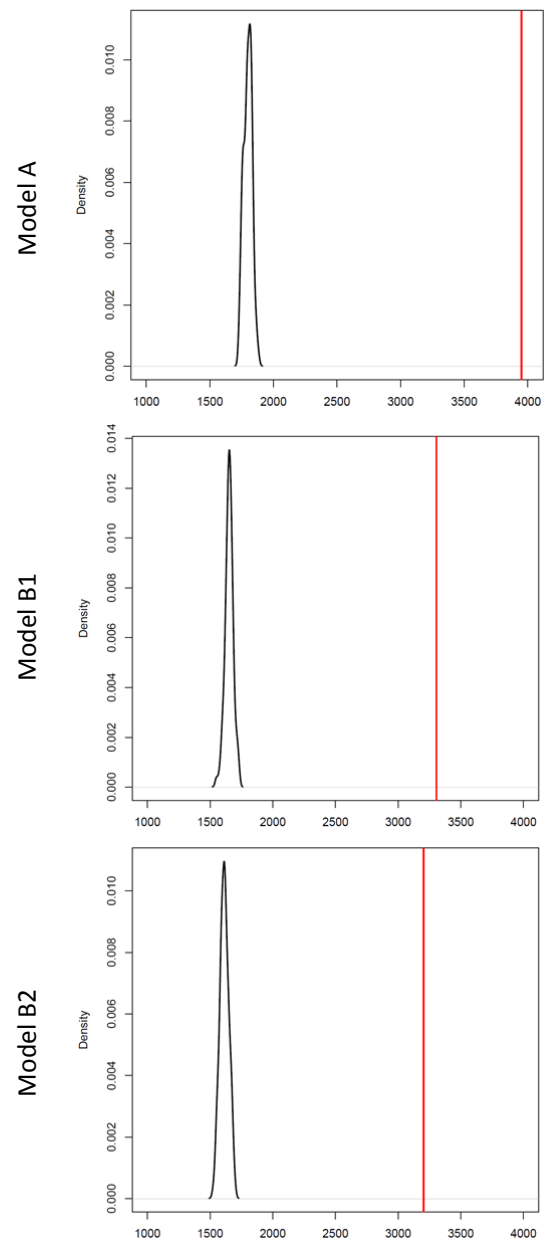

**Figure S12:**

Supplement: Figure S12 — Model tests performed on scenartios of human history shown in Figure 4. The black line represents the distribution of CLRs obtained for data sets generated by parametric bootstraps from simulations done with the maximum composite likelihood parameters obtained for each data set and shown in Table 1. The p-values of each model are then computed as the fraction of data sets with a CLR larger than or equal to the observed CLR (red line), and are zero for all models. Note that the demographic models used for parameter estimations are clearly rejected for both SNP panels, which imply that these models cannot exactly reproduce the observed data. Note however, the shift of the red line to the right for Model B1 and B2, suggesting a much better fit to the data in line with the AIC results shown in Table S2. (PDF) [file pgen.1003905.s012.pdf]

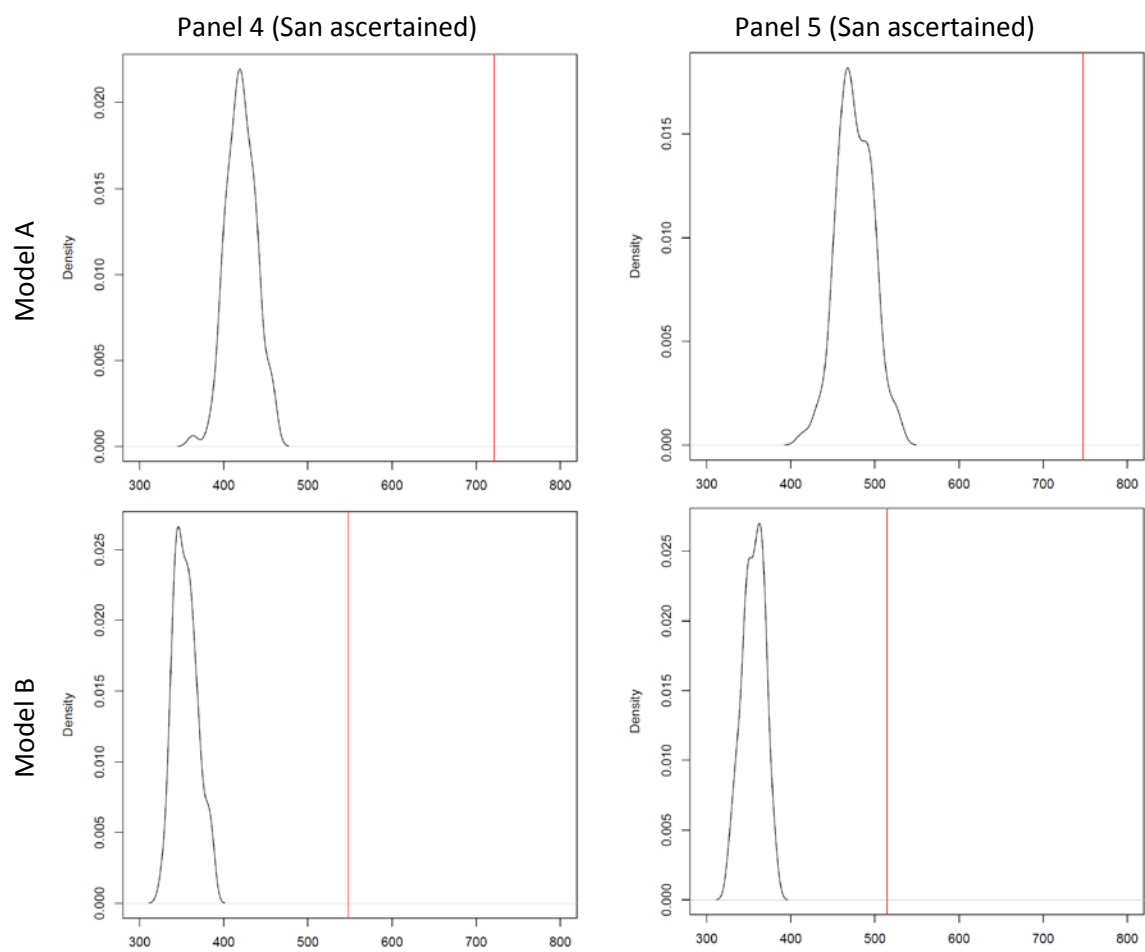

Figure S13:

Supplement: Figure S13 — Model tests performed on scenarios of African history shown in Figure 5 for the San and Yoruba SNP panels. The black line represents the distribution of CLRs obtained for data sets generated by parametric bootstraps from simulations done with the maximum composite likelihood parameters obtained for each data set and shown in Table 2. The p-values of each model are then computed as the fraction of data sets with a CLR larger than or equal to the observed CLR (red line). The demographic models used for parameter estimations are clearly rejected for both SNP panels (p-value = 0 for all models), which imply that these models cannot exactly reproduce the observed data. Note however, the shift of the red line to the right for Model B, suggesting a much better fit to the data in line with the AIC results shown in Table S3. (PDF) [file pgen.1003905.s013.pdf]

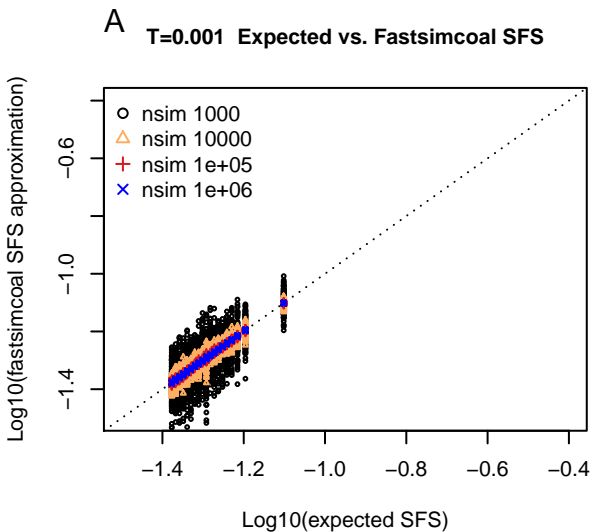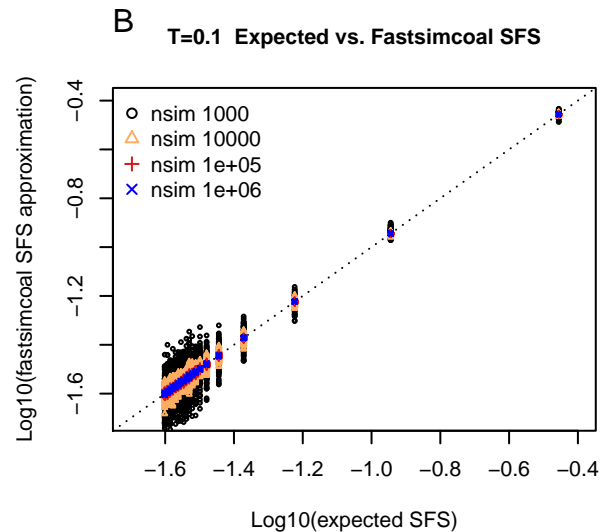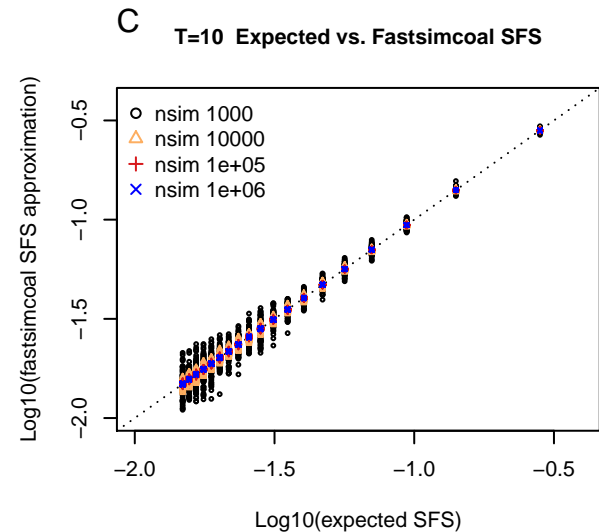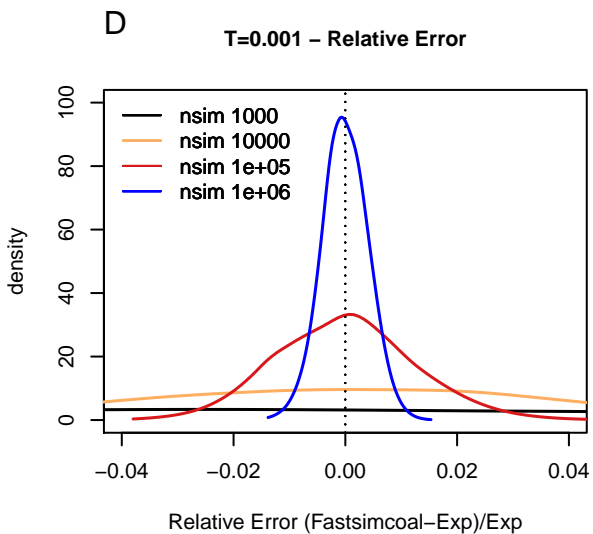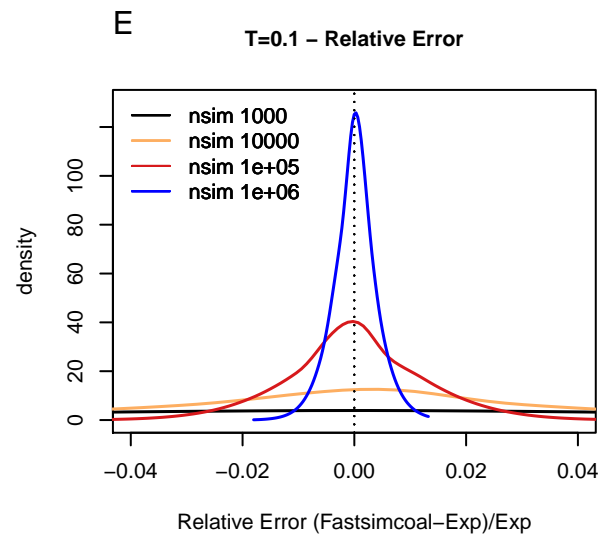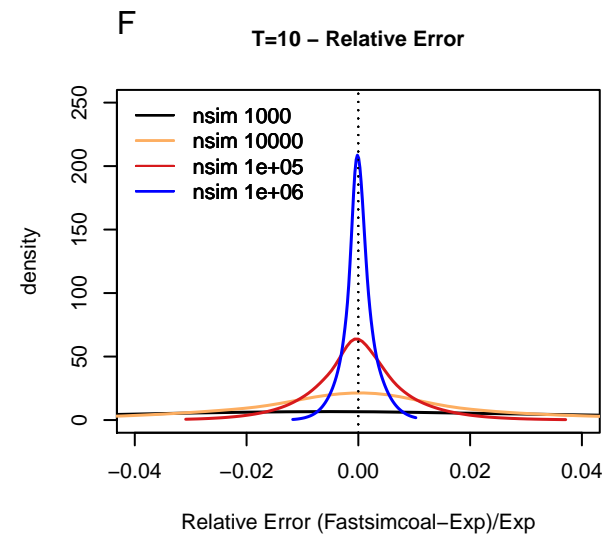

Supplement: Figure S14 — Fit of the SFS approximation obtained with fastsimcoal2 to the expected SFS for a bottleneck model similar to that shown in Fig. 1A. The expected SFS was obtained by implementing eqs. 2–9 from Chen [18] in Mathematica ver 9.0.1.0. We considered a scenario with the following fixed parameter values: 2NCUR = 10000, 2NBOT = 100, and 2NA = 10000. The sample size was fixed to n = 20. We examined the effect of varying the age of the bottleneck TBOT, as 10, 1000, and 10000 generations, which corresponds to a scaled times of 0.001, 0.1, and 1.0 in units of 2NA, and the effect of varying the number of simulations per SFS estimation (between 1 thousand and 1 million). A–C) Direct comparison of the SFS entries (in log scale), where we report fastsimcoal2 results for 100 replicated runs. As can be seen, the higher the number of simulations the better the approximation, as the points get closer to the diagonal. D–F) Relative Error of the fastsimcoal2 SFS approximation, defined as ((SFS fastsimcoal2-exact SFS)/exact SFS). As expected, increasing the number of simulations decreases the relative error. Interestingly, the error distributions are symmetrically distributed around zero suggesting that the fastsimcoal2 approximation is essentially unbiased. (PDF) [file pgen.1003905.s014.pdf]

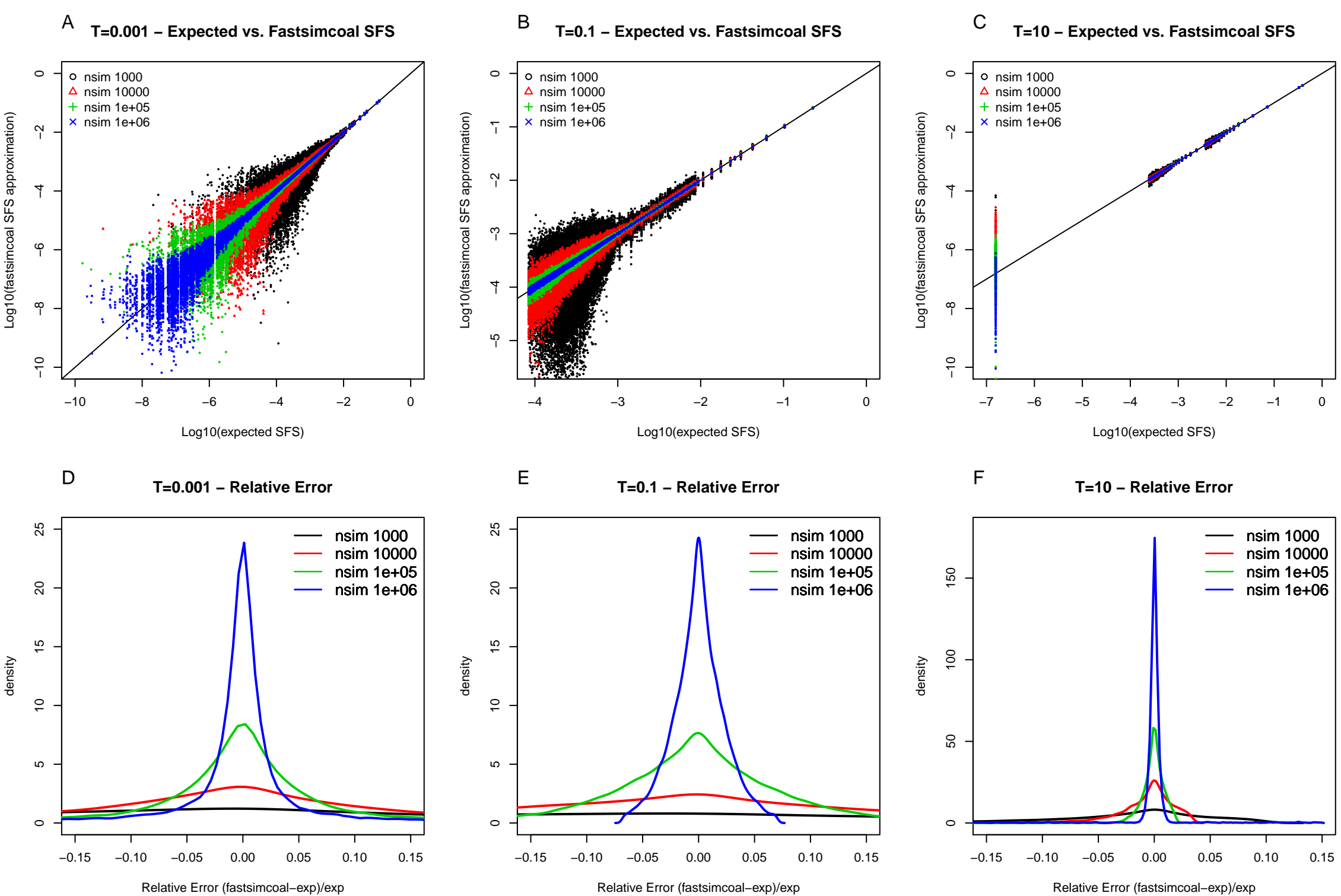

Supplement: Figure S15 — Fit of the SFS approximation obtained with fastsimcoal2 to the expected SFS for a divergence model similar to that in Fig. 1B but without migration. The expected SFS was obtained by implementing eqs. 2–9 from Chen [18] in Mathematica ver 9. We considered a scenario where two populations of different sizes 2N1 = 10000 and 2N2 = 1000 diverged TDIV generations ago from and an ancestral population of size 2NA = 10000. The number of sampled genes are n 1 = 20 and n 2 = 30 for populations 1 and 2, respectively. We examined the effect of using 1 thousand to 1 million simulations to estimate the SFS for varying TDIV values of 0.001, 0.1, and 10, expressed in units of 2NA generations. A–C) Direct comparison of the SFS entries (in log scale), where we report fastsimcoal2 results for 100 replicated runs. Note that we do not show SFS entries estimated by fastsimcoal2 that have a value of zero, but these entries correspond to extremely low expected values. As can be seen, the higher the number of simulations the better the approximation, as the points get closer to the diagonal. D–F) Relative Error of the fastsimcoal2 SFS approximation in log scale. The density curves were obtained after exclusion of outliers (values smaller or larger than the1% and 99% quantiles, respectively). As expected, increasing the number of simulations decreases the relative error. Interestingly, the error distributions are symmetrically distributed around zero suggesting that the fastsimcoal2 approximation is essentially unbiased. (PDF) [file pgen.1003905.s015.pdf]
