# Supplementary material for: Robust Demographic Inference from Genomic and SNP Data
Source: PLoS Genet. 2013 Oct 24;9(10):e1003905. doi: 10.1371/journal.pgen.1003905 (PMC3812088; doi:10.1371/journal.pgen.1003905)
Supplement: Table S1 — Inferred parameters of human demography under model A of Figure 4A. (PDF) [file pgen.1003905.s016.pdf]

Table S1:

Parameters	Point estimation	95% CI Lower bound	Upper bound
N_{ANC}	7963	7810	8610
N_{AFR}	23738	21046	25646
N_{ASW}	171027	34707	232697
N_{CEU}	4106	4029	4321
N_{LWK}	58307	51939	238226
N_{YRI}	4038	3388	12489
T_{NC}	70	51	208
a_E	0.16	0.15	0.18
a_Y	0.84	0.77	0.85
T_{BOT}	2683	2386	3718
N_{BOT}	7083	3219	10758
