# Supplementary material for: Robust Demographic Inference from Genomic and SNP Data
Source: PLoS Genet. 2013 Oct 24;9(10):e1003905. doi: 10.1371/journal.pgen.1003905 (PMC3812088; doi:10.1371/journal.pgen.1003905)
Supplement: Table S2 — Relative likelihood of the different models shown in Figure 4. (PDF) [file pgen.1003905.s017.pdf]

**Table S2:**

| Model | Max $\log_{10}(Lhood_i)$ [a] | No. of parameters (*d*) | $AIC_i$ [b] | $\Delta_i$ [c] | Model normalized relative likelihood ($w_i$) [d] |
|---|---|---|---|---|---|
| A | -533989 | 12 | 2459477 | 3535.15 | ~0 |
| B1 | -533317 | 16 | 2456442 | 468.601 | $2.8\times10^{-95}$ |
| B2 | -533210 | 19 | 2456007 | 0 | ~1 |

[a]Based on the best of 100 likelihood computed for parameters point estimates shown in Table 1 and S1.

[b] $AIC_i = 2d - 2\ln(Lhood_i)$

[c] $\Delta_i = AIC_i - \min(AIC)$

[d] $w_i = \dfrac{\exp(-0.5\Delta_i)}{\sum_r^R \exp(-0.5\Delta_r)}$
