# Supplementary material for: Robust Demographic Inference from Genomic and SNP Data
Source: PLoS Genet. 2013 Oct 24;9(10):e1003905. doi: 10.1371/journal.pgen.1003905 (PMC3812088; doi:10.1371/journal.pgen.1003905)
Supplement: Table S3 — Relative likelihood of the different models shown in Figure 5 for SNP chip panels 4 and 5. (PDF) [file pgen.1003905.s018.pdf]

Table S3:

SNP chip panel	Model	Max $\log_{10}(Lhood_i)$ [a]	No. of parameters (*d*)	AIC_i [b]	Δ_i [c]	Model normalized relative likelihood (w_i) [d]
4 (San)	A	-261372	12	1204190	415.508	5.96E-91
	B	-261314	16	1203774	0	1
5 (Yoruba)	A	-201248	12	927282	416.8989	2.967E-91
	B	-201189	16	926865	0	1

[a]Based on the best of 100 likelihood computed for parameters point estimates shown in Table 2.

[b] $AIC_i = 2d - 2\ln(Lhood_i)$

[c] $\Delta_i = AIC_i - \min(AIC)$

[d] $w_i = \frac{\exp(-0.5\Delta_i)}{\sum_r^R \exp(-0.5\Delta_r)}$
