# Supplementary material for: Robust Demographic Inference from Genomic and SNP Data
Source: PLoS Genet. 2013 Oct 24;9(10):e1003905. doi: 10.1371/journal.pgen.1003905 (PMC3812088; doi:10.1371/journal.pgen.1003905)
Supplement: Text S1 — Input file format and fastsimcoal2 command lines. (PDF) [file pgen.1003905.s019.pdf]

Input file format and *fastsimcoal2* command lines

Input files

Parameter estimation with fastsimcoal2 requires three input files and specific command lines parameterizing the estimation. The first input files is a template file (usually with a *.tpl extension) specifying the sample sizes, the sampling times, as well as the demographic model.

fastsimcoal2 model specification follows exactly the same syntax as *simcoal2* (http://cmpg.unibe.ch/software/simcoal2) and *fastsimcoal* (http://cmpg.unibe.ch/software/fastsimcoal), but the parameters of interest are replaced by keywords. In addition, a new data type FREQ needs to be specified for parameter estimation from SFS (see example tpl files in the SI). The second input file specifies search ranges for the parameters to be estimated, and usually has a *.est extension. The syntax is similar to that used for .est files in ABCToolBox (Wegmann et al. 2010), but with some notable differences: 1) minimum and maximum parameter values are not boundaries of a prior distribution, but limits of parameter search ranges. Note that for fastsimcoal2 the lower bound of the search interval is fixed, whereas the upper bound is only fixed for choosing the initial parameter values and therefore the parameter space can be explored beyond this initial upper limit. The third input file contains the observed site frequency spectrum (SFS) on a single line, using the same format as in $\partial a \partial i$ (see example file p4NocpgSanYor4_DSFS-asc2.obs in SI). For more than three, say *n*, populations, one can perform estimations from multiple pairwise joint SFS, and in this case $n(n-1)/2$ joint SFS files are needed. The name of these input files needs to be the same as that of the *tpl* file, followed by the suffix *jointDAFPop_i_j* (where $i>j$). For instance, for a tpl file called testFS.tpl, and a model with 3 populations, the following files would be needed: testFS_jointDAFpop1_0.obs, testFS_jointDAFpop2_1.obs, and testFS_jointDAFpop2_0.obs. An example of such a joint SFs file (IM20Mb_jointDAFpop1_0.obs) is presented in the SI.

Command line arguments

The following command line parameters need to be input for parameter estimations: Name of tpl file (-t), name of est file (-e), number of simulations for estimation of expected SFS (-n), estimation from the unfolded (-d) or folded (-m), minimum number of estimation rounds (-l), maximum number of estimation rounds (-L), criterion for convergence (-M), an optional seed (--seed), a flag for the estimation from the joint multidimensional SFS (--multiSFS), optional quiet mode flag (-q), minimum count of observed SFS entries entering in the likelihood computation(-C) (the smaller entries are collapsed in a single category for the likelihood computation). In case of ascertained data, the additional two options are necessary: ascertained population (-a), number of genes in the ascertainment panel (-A). Note that in case of estimations from ascertained SNP panels, the observed SFS has the suffix "-ascx.obs" instead of ".obs" SNPs inferred from DNA sequence data, where x is the ascertainment panel size (i.e. x=2 for the Affymetrix chip panels).
