# Supplementary material for: Robust Demographic Inference from Genomic and SNP Data
Source: PLoS Genet. 2013 Oct 24;9(10):e1003905. doi: 10.1371/journal.pgen.1003905 (PMC3812088; doi:10.1371/journal.pgen.1003905)
Supplement: Text S2 — Examples of input files used in the paper. (PDF) [file pgen.1003905.s020.pdf]

Examples of input files used in the paper

Input files for the bottleneck scenario in Figure1A

1PopBot20Mb.tpl file

```
//Number of population samples (demes)
1
//Population effective sizes (number of genes)
NCUR
//Sample sizes
20
//Growth rates : negative growth implies population expansion
0
//Number of migration matrices : 0 implies no migration between demes
0
//historical event: time, source, sink, migrants, new size, new growth rate, migr. matrix
2  historical event
TBOT 0 0 0 RESBOT 0 0
TENDBOT 0 0 0 RESENDBOT 0 0
//Number of independent loci [chromosome]
1 0
//Per chromosome: Number of linkage blocks
1
//per Block: data type, num loci, rec. rate and mut rate + optional parameters
FREQ 1 0 2.5e-8
```

1PopBot20Mb.est file

```
// Priors and rules file
// *********************

[PARAMETERS]
//#isInt? #name   #dist.#min  #max
//all Ns are in number of haploid individuals
1  NCUR  unif 10  100000 output
1  NANC  unif 10  100000 output
1  NBOT  unif 10  100000 output
1  TBOT  unif 10  10000 output

[RULES]

[COMPLEX PARAMETERS]
0 RESBOT    = NBOT/NCUR hide
0 RESENDBOT = NANC/NBOT hide
1 TENDBOT   = TBOT+100  hide
```

Command line for parameter estimation

```
./fastsimcoal2 -t 1PopBot20Mb.tpl -n100000 -N100000 -d -e 1PopBot20Mb.est -M 0.001 –l 10 -L 40
–q
```

Example of an observed SFS file

1PopBot20Mb_DAFpop0.obs

```
1 observations
d0_0      d0_1 d0_2  d0_3  d0_4  d0_5  d0_6  d0_7  d0_8  d0_9  d0_10 d0_11 d0_12 d0_13 d0_14 d0_15 d0_16 d0_17 d0_18 d0_19 d0_20
19960052  9331 3572  2530  2221  2059  1963  1952  1730  1682  1572  1520  1426  1453  1335  1179  1195  1069  1129  1030  0
```

Input files for the IM scenario in Figure 1B

IM20Mb.tpl file

```
//Parameters for the coalescence simulation program : simcoal.exe
2 samples to simulate :
//Population effective sizes (number of genes)
NPOP1
NPOP2
//Samples sizes and samples age
20
30
//Growth rates: negative growth implies population expansion
0
0
//Number of migration matrices : 0 implies no migration between demes
2
//Migration matrix 0
0 MIG2
MIG1 0
//Migration matrix 1
0 0
0 0
//historical event: time, source, sink, migrants, new deme size, growth rate, migr mat index
1 historical event
TDIV 0 1 1 RESIZE 0 1
//Number of independent loci [chromosome]
1 0
//Per chromosome: Number of contiguous linkage Block: a block is a set of contiguous loci
1
//per Block:data type, number of loci, per gen recomb and mut rates
FREQ 1 0 2.5e-8
```

IM20Mb.est file

```
// Priors and rules file
// *********************

[PARAMETERS]
//#isInt? #name   #dist.#min  #max
//all N are in number of haploid individuals
1  ANCSIZE     unif     100  100000   output
1  NPOP1       unif     100  100000   output
1  NPOP2       unif     100  100000   output
0  N1M1        logunif  1e-2 20       output
0  N2M2        logunif  1e-2 20       output
1  TDIV        unif     100   20000   output

[RULES]

[COMPLEX PARAMETERS]

0  2NM1 = 2*N1M1              hide
0  2NM2 = 2*N2M2              hide
0  RESIZE = ANCSIZE/NPOP2     hide
0  MIG1    = 2NM1/NPOP1       hide
0  MIG2    = 2NM2/NPOP2       hide
```

Command line for parameter estimation

```
./fastsimcoal2 -t IM20Mb.tpl -n100000 -N100000 -d -e IM20Mb.est -M 0.001 –l 10 -L 40 -q
```

Example of a joint observed SFS file

IM20Mb_jointDAFpop1_0.obs

1 observations
1 observation

	d0_0	d0_1	d0_2	d0_3	d0_4	d0_5	d0_6	d0_7	d0_8	d0_9	d0_10	d0_11	d0_12	d0_13	d0_14	d0_15	d0_16	d0_17	d0_18	d0_19	d0_20
d1_0	19928211	15071	5514	2678	1397	799	498	259	173	83	54	27	16	2	3	1	1	0	0	0	0
d1_1	2103	646	481	385	282	211	183	116	70	47	42	16	12	11	3	0	1	0	0	0	0
d1_2	900	515	386	313	240	171	145	123	81	38	38	25	14	11	5	2	3	1	0	0	0
d1_3	537	397	345	291	229	192	151	115	89	59	41	26	21	12	8	3	2	0	0	0	0
d1_4	349	317	318	284	229	199	147	119	95	62	30	29	22	15	10	7	1	2	0	0	0
d1_5	284	237	256	252	230	202	166	103	95	63	63	36	30	13	11	4	2	3	1	0	0
d1_6	205	223	246	230	203	187	150	121	102	77	55	42	34	23	12	8	3	0	0	0	0
d1_7	130	196	213	201	202	184	155	138	117	94	56	41	32	21	11	9	4	1	1	0	0
d1_8	109	160	188	194	169	184	139	128	106	82	55	52	40	22	14	16	5	1	1	0	0
d1_9	68	118	123	189	158	167	148	128	101	103	74	55	39	36	19	13	13	5	0	1	0
d1_10	46	86	150	167	168	149	138	118	102	68	67	48	59	23	28	13	9	6	2	1	0
d1_11	39	79	108	129	155	151	162	115	122	78	83	72	60	32	39	21	13	10	1	1	0
d1_12	26	68	91	135	141	131	118	119	106	106	92	51	38	39	29	28	11	7	4	1	1
d1_13	25	39	67	95	111	113	141	112	94	120	101	85	73	55	39	15	23	16	4	0	0
d1_14	16	34	73	89	100	110	105	112	95	94	74	57	62	49	54	33	23	9	7	4	0
d1_15	5	30	43	62	96	93	102	118	100	108	89	68	55	44	40	32	25	12	8	7	1
d1_16	8	19	46	60	81	95	89	108	99	90	79	71	68	71	49	41	20	16	17	9	2
d1_17	3	14	34	42	63	77	84	102	86	99	101	85	65	55	52	29	23	20	14	6	3
d1_18	3	13	35	50	49	79	87	83	104	90	93	72	73	69	55	30	29	24	15	10	4
d1_19	2	8	19	44	47	71	63	80	84	91	78	68	78	60	60	51	30	35	14	12	1
d1_20	2	9	15	33	42	57	64	62	82	77	66	77	92	63	40	45	41	25	22	9	0
d1_21	3	8	15	25	33	41	59	63	79	75	72	82	80	76	60	51	50	20	25	10	1
d1_22	1	5	12	15	29	27	41	44	64	71	89	71	73	66	70	61	40	34	29	15	7
d1_23	0	5	3	13	27	21	43	55	71	54	63	82	75	88	70	70	53	36	35	19	3
d1_24	0	0	6	13	19	25	33	45	52	52	48	50	64	73	86	58	55	47	38	23	12
d1_25	1	1	2	9	11	29	18	33	40	59	65	65	81	72	56	54	63	59	35	19	4
d1_26	0	2	0	8	8	16	15	32	41	48	60	59	59	73	68	59	69	57	38	30	21
d1_27	0	0	2	3	8	10	13	20	30	42	63	63	61	65	68	68	72	71	47	44	19
d1_28	0	0	0	2	5	3	11	19	25	28	38	41	69	64	64	71	90	82	67	57	31
d1_29	0	0	3	2	3	3	7	10	23	40	45	55	53	70	75	75	70	91	80	49	54
d1_30	0	0	0	2	4	4	7	14	29	37	60	85	105	183	201	312	371	490	638	830	0

Input files for the 3-population scenario in Figure 1C

3PopExpBot20Mb.tpl

```
//Parameters for the coalescence simulation program : fastsimcoal.exe
3 samples to simulate :
//Population effective sizes (number of genes)
NPOPAF
2000000
2000000
//Samples sizes and samples age
20
20
20
//Growth rates : negative growth implies population expansion
0
R1
R1
//Number of migration matrices : 0 implies no migration between demes
2
//Migration matrix 0
0.0000 0.0000 0.0000
0.0000 0.0000 MIG
0.0000 MIG 0.0000
//Migration matrix 1
0 0 0
0 0 0
0 0 0
//historical event: time, source, sink, migrants, new deme size, growth rate, migr mat index
4 historical event
TDIV 2 0 1 1 0 1
TDIV 1 0 1 1 0 1
TBOT 0 0 0 RES1 0 1
TENDBOT 0 0 0 RES2 0 1
//Number of independent loci [chromosome]
1 0
//Per chromosome: Number of contiguous linkage Block: a block is a set of contiguous loci
1
//per Block:data type, number of loci, per gen recomb and mut rates
FREQ 1 0 2.5e-8
```

3PopExpBot20Mb.est

```
// Priors and rules file
// *********************

[PARAMETERS]
//#isInt? #name   #dist.#min  #max
//all Ns are in number of haploid individuals
1  ANCSIZE     unif     1000  100000  output
1  NBOT        unif     10   2000     output
1  NPOPAF      unif     1000 100000   output
1  NPOPOOA     unif     10   10000    output
1  TDIV        unif     10   10000    output
1  TPLUSDIV    unif     10   10000    hide
0  MIG         logunif  1e-5 1e-2     output

[RULES]

[COMPLEX PARAMETERS]
1  TBOT = TDIV+TPLUSDIV           output
0  RATIO_OOA_EA = NPOPOOA/2000000 hide
0  RTEA = log(RATIO_OOA_EA)       hide
0  R1 = RTEA/TDIV                 hide
1  TENDBOT = TBOT+500             hide
0  RES1 = NBOT/NPOPAF             hide
0  RES2 = ANCSIZE/NBOT            hide
```

Command line for parameter estimation

```
./fastsimcoal2 -t 3PopExpBot20Mb.tpl -n100000 -N100000 -d -e 3PopExpBot20Mb.est -M 0.001 –l 10
-L 40 -q --multiSFS -C10
```

Input files for the 10-population scenario in Figure 1D

10Pop2ContiIsl.tpl

```
//Parameters for the coalescence simulation program : fastsimcoal.exe
12 samples to simulate :
//Population effective sizes (number of genes)
1000
1000
1000
1000
1000
1000
1000
1000
1000
1000
20000000
20000000
//Samples sizes and samples age
20
20
20
20
20
20
20
20
20
20
0
0
//Growth rates   : negative growth implies population expansion
0
0
0
0
0
0
0
0
0
0
0
0
//Number of migration matrices : 0 implies no migration between demes
3
//Migration matrix 0
0	0	0	0	0	0	0	0	0	0	M010	0
0	0	0	0	0	0	0	0	0	0	M110	0
0	0	0	0	0	0	0	0	0	0	M210	0
0	0	0	0	0	0	0	0	0	0	M310	0
0	0	0	0	0	0	0	0	0	0	M410	0
0	0	0	0	0	0	0	0	0	0	0	M511
0	0	0	0	0	0	0	0	0	0	0	M611
0	0	0	0	0	0	0	0	0	0	0	M711
0	0	0	0	0	0	0	0	0	0	0	M811
0	0	0	0	0	0	0	0	0	0	0	M911
0	0	0	0	0	0	0	0	0	0	0	0
0	0	0	0	0	0	0	0	0	0	0	0
//Migration matrix 1
0	0	0	0	0	0	0	0	0	0	0	0
0	0	0	0	0	0	0	0	0	0	0	0
0	0	0	0	0	0	0	0	0	0	0	0
0	0	0	0	0	0	0	0	0	0	0	0
0	0	0	0	0	0	0	0	0	0	0	0
0	0	0	0	0	0	0	0	0	0	0	M511
0	0	0	0	0	0	0	0	0	0	0	M611
0	0	0	0	0	0	0	0	0	0	0	M711
0	0	0	0	0	0	0	0	0	0	0	M811
0	0	0	0	0	0	0	0	0	0	0	M911
0	0	0	0	0	0	0	0	0	0	0	M1211
0	0	0	0	0	0	0	0	0	0	0	0
//Migration matrix 2
0	0	0	0	0	0	0	0	0	0	0	0
0	0	0	0	0	0	0	0	0	0	0	0
0	0	0	0	0	0	0	0	0	0	0	0
0	0	0	0	0	0	0	0	0	0	0	0
0	0	0	0	0	0	0	0	0	0	0	0
0	0	0	0	0	0	0	0	0	0	0	0
0	0	0	0	0	0	0	0	0	0	0	0
0	0	0	0	0	0	0	0	0	0	0	0
0	0	0	0	0	0	0	0	0	0	0	0
0	0	0	0	0	0	0	0	0	0	0	0
0	0	0	0	0	0	0	0	0	0	0	0
0	0	0	0	0	0	0	0	0	0	0	0
//historical event: time, source, sink, migrants, new deme size, new growth rate, migration matrix index
```

```
13 historical event
TISLAND1 0 10 1 1 0 1
TISLAND1 1 10 1 1 0 1
TISLAND1 2 10 1 1 0 1
TISLAND1 3 10 1 1 0 1
TISLAND1 4 10 1 1 0 1
TISLAND1 10 10 0 0.0001 0 1
TISLAND2 5 11 1 1 0 2
TISLAND2 6 11 1 1 0 2
TISLAND2 7 11 1 1 0 2
TISLAND2 8 11 1 1 0 2
TISLAND2 9 11 1 1 0 2
TISLAND2 10 11 1 1 0 2
TISLAND2 11 11 0 RESIZE 0 2//Number of independent loci [chromosome]
1 0
//Per chromosome: Number of contiguous linkage Block: a block is a set of contiguous loci
1
//per Block:data type, number of loci, per gen recomb and mut rates
FREQ 1 0 2.5e-8
```

10Pop2ContiIsl.est

```
// Priors and rules file
// *********************

[PARAMETERS]
//#isInt? #name   #dist.#min  #max
//all Ns are in number of haploid individuals
1  ANCSIZE     unif     10  100000    output
0  NM0         logunif  0.01  100     output
0  NM1         logunif  0.01  100     output
0  NM2         logunif  0.01  100     output
0  NM3         logunif  0.01  100     output
0  NM4         logunif  0.01  100     output
0  NM5         logunif  0.01  100     output
0  NM6         logunif  0.01  100     output
0  NM7         logunif  0.01  100     output
0  NM8         logunif  0.01  100     output
0  NM9         logunif  0.01  100     output
0  NM_12        logunif  0.01  100    output
1  TISLAND1    unif     10    20000   output
1  TPLUS       unif     10    20000   hide

[RULES]

[COMPLEX PARAMETERS]

//Assume an island haploid population size of 1000 for all islands
1  TISLAND2 = TISLAND1+TPLUS output
0  M010 =  NM0/1000          hide
0  M110 =  NM1/1000          hide
0  M210 =  NM2/1000          hide
0  M310 =  NM3/1000          hide
0  M410 =  NM4/1000          hide
0  M511 =  NM5/1000          hide
0  M611 =  NM6/1000          hide
0  M711 =  NM7/1000          hide
0  M811 =  NM8/1000          hide
0  M911 =  NM9/1000          hide
0  M1211 = NM_12/2000        hide
0  RESIZE = ANCSIZE/20000000 hide
```

Command line for parameter estimation

```
./fastsimcoal2 -t 10Pop2ContiIsl.tpl -n50000 -N50000 -d -e 10Pop2ContiIsl.est -M 0.001 - l10 –
L 30 -q
```

Input files for the African demography with SNP ascertainment as in Figure 5

Model A

p4NocpgSanYor4.tpl

```
//Parameters for the coalescence simulation program : simcoal.exe
3 samples to simulate : Exponential growth : 1000 to 100,000,000 started 3000 generations ago
//Population effective sizes (number of genes)
NSan
NYor
1000
//Samples sizes
12
44
1 1600
//Growth rates   : negative growth implies population expansion
0
0
0
//Number of migration matrices : 0 implies no migration between demes
0
//historical event: time, source, sink, migrants, new deme size, new growth rate, migration matrix index
6  historical event
TGSan 0 0 0 RES_SAN 0 0
TGYor 1 1 0 RES_YOR 0 0
TAdm 0 1 AYS 1 0 0
TAdm 1 0 ASY 1 0 0
TDIVSanYor 1 0 1 RES_AF 0 0
16000 2 0 1 RES_ANC 0 0
//Number of independent loci [chromosome]
1 0
//Per chromosome: Number of contiguous linkage Block: a block is a set of contiguous loci
1
//per Block:data type, number of loci, per gen recomb and mut rates
FREQ 1 0 1e-7
```

p4NocpgSanYor4.est

```
// Priors and rules file
// *********************

[PARAMETERS]
//#isInt? #name   #dist.#min  #max
//all Ns are in number of haploid individuals
1  NSan        unif     1000   2e6  output
1  NYor        unif     1000   2e6  output
1  NASan       unif     1000   1e5  output
1  NAYor       unif     1000   1e5  output
1  HSIZE       unif     1000   1e5  output
1  ANCSIZE     unif     1000   1e5  output
1  TGSan       unif     10     500  output
1  TGYor       unif     10     500  output
1  TAdm        unif     10     500  output
1  TPlusAdm    unif     1     5000  hide
0  AYS         unif     0      0.2  output
0  ASY         unif     0      0.2  output

[RULES]

[COMPLEX PARAMETERS]
1  TDIVSanYor = TAdm+TPlusAdm   output
0  RES_SAN  = NASan/NSan        hide
0  RES_YOR  = NAYor/NYor        hide
0  RES_AF   = HSIZE/NASan       hide
0  RES_ANC  = ANCSIZE/HSIZE     hide
```

Command line for parameter estimation

./fastsimcoal2 -t p4NocpgSanYor4.tpl -n100000 -N100000 -d -e p4NocpgSanYor4.est -M 0.001 –l 10 -L 20 -a0 -A2 -q -0 -C2 –multiSFS

Model B

p4NocpgSanYor4.tpl

```
//Parameters for the coalescence simulation program : simcoal.exe
5 samples to simulate : Exponential growth : 1000 to 100,000,000 started 3000 generations ago
//Population effective sizes: San, Yoruba, Cont San, Cont Yor, Denisova
500
500
1000
NCSan
NCYor
//Samples sizes
12
44
1 1600
0
0
//Growth rates   : negative growth implies population expansion
0
0
0
RSan
RYor
//Number of migration matrices : 0 implies no migration between demes
3
//Current migration matrix 0
0       0       0       mS      0
0       0       0       0       mY
0       0       0       0       0
0       0       0       0       0
0       0       0       0       0
//Current migration matrix 1
0       0       0       mS      0
0       0       0       0       0
0       0       0       0       0
0       0       0       0       m_YS
0       0       0       m_SY    0
//No migration matrix 2
0       0       0       0       0
0       0       0       0       0
0       0       0       0       0
0       0       0       0       0
0       0       0       0       0
//historical event: time, source, sink, migrants, new deme size, new growth rate, migration matrix index
8  historical event
TAdm 3 4 a_YS 1 RYor 0
TAdm 4 3 a_SY 1 RSan 0
TEY 1 4 1 RESYOR 0 1
TES 3 3 0 1 0 1
TES 0 4 1 RESNH 0 2
TES 1 4 1 1 0 2
TES 3 4 1 1 0 2
16000 2 4 1 RESANC 0 2
//Number of independent loci [chromosome]
1 0
//Per chromosome: Number of contiguous linkage Block: a block is a set of contiguous loci
1
//per Block:data type, number of loci, per generation recombination and mutation rates and optional
parameters
FREQ 1 0 2.5e-8 OUTEXP
```

p4NocpgSanYor4.est

```
// Priors and rules file
// *********************

[PARAMETERS]
//#isInt? #name   #dist.#min  #max
//all Ns are in number of haploid individuals
1  ANCSIZE     unif     1000    100000   output
1  HSIZE       unif     1000    100000   output
1  NAYor       unif     1000    100000   output
1  NDivSan     unif     100      10000   output
1  NDivYor     unif     100      10000   output
1  NCYor       unif     1000   1000000   output
1  NCSan       unif     1000   1000000   output
0  Nm_S        unif     0          100   output
0  Nm_Y        unif     0          100   output
1  TAdm        unif     0          100   output
0  a_YS        unif     0          0.2   output
0  a_SY        unif     0          0.2   output
0  m_SY        logunif  1e-8      1e-3   output
0  m_YS        logunif  1e-8      1e-3   output
1  TExtraGrowth unif    1          500   hide
1  TExtraDiv   unif     1         5000   hide

[RULES]


[COMPLEX PARAMETERS]

1  TEY      = TAdm+TExtraGrowth output
1  TES      = TEY+TExtraDiv     output

//Computation of Yoruba growth rate
0  RatioYor = NDivYor/NCYor   hide
0  logRYor  = log(RatioYor)   hide
0  RYor     = logRYor/TEY     hide

//Computation of San growth rate
0  RatioSan = NDivSan/NCSan   hide
0  logRSan  = log(RatioSan)   hide
0  RSan     = logRSan/TES     hide

0  mS       = Nm_S/500      hide
0  mY       = Nm_Y/500        hide

0  RESYOR   = NAYor/NDivYor   hide
0  RESNH    = HSIZE/NAYor     hide
0  RESANC   = ANCSIZE/HSIZE   hide
```

Observed joint multidimensional SFS in San, Yoruba and Denisova for Affymetrix panel 4

p4NocpgSanYor4_DSFS-asc2.obs (the multidimensional SFS is given on a single line) using the same representation as $\partial a \partial i$.

```
1 observation. No. of demes and sample sizes are on next line
3   12    44    1
0    0     0    0    0    0    0    0    0    0    0    0    0     0    0    0    0    0    0
     0     0    0    0    0    0    0    0    0    0    0    0     0    0    0    0    0    0
     0     0    0    0    0    0    0    0    0    0    0    0     0    0    0    0    0    0
     0     0    0    0    0    0    0    0    0    0    0    0     0    0    0    0    0    0
     0     0    0    0    0    0    0    0    0    0    0    0     0    0    0    0    0
 11407   127 1701   71 1237   55 1096   60  829   65  662   44   600   26  510   29  442   29
   388    50  325   28  291   34  271   21  192   18  198   21   173   18  114   12  106   13
    95    10   93   14   84   10   88    8   43    4   44   10    34    8   39    6   30    6
    23     3   24    4   18    2   12    1   10    4    8    4     9    1    6    0    5    4
     4     3    5    2    1    0    1    0    0    0    1    0     0    0    0    1    0    0
  7796   148 1454   77 1111   85  917   75  819   56  757   67   603   63  584   50  507   68
   509    52  420   46  383   37  337   33  302   46  242   36   239   44  227   45  200   35
   191    29  147   20  140   25  117   24  128   30  107   17    92   18   76   11   68   14
    74    17   36   14   32   10   38    8   37   10   31    9    26    5   21    5   13    6
     7     2   12   11   11    1    3    1    5    3    2    0     1    2    0    2    2    0
  4478   115  993   68  815   64  661   65  663   47  563   68   548   53  449   63  477   59
   377    65  390   53  326   46  296   46  307   54  288   50   279   36  226   45  221   36
   225    38  191   28  188   41  184   41  152   32  145   49   107   24  107   33   98   35
    88    21   75   37   58   20   51   22   55   32   46   15    33   11   36   15   22   10
    24     7   21   14   12    6    8    9    9    4    8    4     1    7    1    1    3    6
  2194    65  699   55  563   50  478   46  459   54  447   54   404   53  380   62  387   64
   321    57  303   67  278   68  334   67  253   51  237   64   256   43  245   39  217   55
   220    66  210   48  179   44  192   46  170   51  144   41   129   31  129   42   94   43
   104    28   98   42   79   31   92   26   58   33   53   25    59   23   50   17   40   18
    34    22   38   15   22   20   19    7   15   12   13    9    16    6   12    5    4    8
  1117    41  371   36  333   39  307   43  299   41  288   53   255   60  283   60  265   47
   254    57  245   49  248   53  245   64  216   59  207   56   209   70  210   67  220   60
   193    47  227   61  176   58  176   50  184   58  140   45   142   66  149   50  124   55
   118    37  107   55   95   43  106   38   87   40   68   50    64   38   59   33   59   38
    40    29   57   31   35   30   30   26   33   12   13   12    14   20   15    6   15   14
   495    17  198   15  206   27  195   26  207   26  165   29   167   27  159   42  179   52
   210    58  158   36  170   36  171   66  174   55  185   43   183   39  173   53  175   47
   171    57  162   65  138   70  148   54  142   43  123   63   142   59  126   51  108   74
   112    60  117   61   70   61   78   44   92   51  100   52    77   51   83   36   62   46
    75    44   40   44   32   38   52   28   33   34   29   23    18   20   14   20   15   19
   204    10  100   20  123   16   95   16  107   18  101   22   113   23  103   27  117   26
   107    18  115   46  128   43  120   35  123   45  138   45   124   46  120   47  143   33
   109    38  134   49  107   52  130   48  120   56  125   66    99   55  103   69   97   61
   100    69  108   58  106   54   91   48  100   60   88   49    96   60   73   71   86   61
    68    56   60   56   73   59   42   53   61   36   41   33    37   38   29   40   28   40
    92     6   60    8   48    7   51    5   45   18   64   11    55   19   66   18   68   16
    68    13   72   20   84   20   77   22   76   30   86   32    75   32   93   42   92   41
    89    43   83   51   85   47   91   49   91   53   71   52    78   58  102   35  106   57
    92    57   79   62   85   59   88   64   84   56   98   68    79   55   70   61   87   68
    73    82   60   59   60   66   66   65   62   61   63   56    41   47   48   52   60   91
    35     1   18    1   24    3   27    3   27    7   31    9    25   10   33    8   35    8
    34    11   41    9   38   14   48   17   34   27   46   17    31   28   57   17   45   20
    60    28   47   29   54   49   66   41   72   34   66   35    46   56   57   36   81   42
    69    47   71   57   63   54   85   42   67   45   77   44    66   47   61   64   43   63
    69    73   58   63   61   69   68   75   61   47   60   55    52   78   54   77   45  113
     9     2    7    1    5    3    9    0    6    2    6    1     6    1   19    2    6    9
    18     3   13    5   25    5   15   11    9    4   14   14    13   10   22    8   36   23
    26     9   23   27   30   22   22   18   30   21   26   20    32   35   41   22   33   35
    43    35   52   43   39   35   44   35   55   27   48   50    61   44   44   45   47   67
    53    65   45   78   63   58   52   66   64   83   56   73    49   87   63   81   84  232
     1     1    1    0    0    0    2    0    3    3    1    1     2    0    5    1    1    3
     2     0    4    4    3    1    4    2    4    1    4    0     4    3    7    2    6   11
     6     4    6    5    6    9    7   11   13    2   11   10    12    3   25   10   10   15
    16    11   26   10   21   20   14   17   22   27   23   21    32   40   22   22   31   28
    27    31   20   32   44   55   33   53   41   53   42   66    44   64   54   84   88  200
     0     0    0    0    0    0    0    0    0    0    0    0     0    0    0    0    0    0
     0     0    0    0    0    0    0    0    0    0    0    0     0    0    0    0    0    0
     0     0    0    0    0    0    0    0    0    0    0    0     0    0    0    0    0    0
     0     0    0    0    0    0    0    0    0    0    0    0     0    0    0    0    0    0
     0     0    0    0    0    0    0    0    0    0    0    0     0    0    0    0    0    0
```
